# Emergence of Azithromycin-Resistant *Neisseria gonorrhoeae* Isolates Belonging to the NG-MAST Genogroup 12302 in Russia

**DOI:** 10.3390/microorganisms11051226

**Published:** 2023-05-06

**Authors:** Ilya Kandinov, Ekaterina Dementieva, Marina Filippova, Alexandra Vinokurova, Sofya Gorshkova, Alexey Kubanov, Victoria Solomka, Julia Shagabieva, Dmitry Deryabin, Boris Shaskolskiy, Dmitry Gryadunov

**Affiliations:** 1 Center for Precision Genome Editing and Genetic Technologies for Biomedicine, Engelhardt Institute of Molecular Biology, Russian Academy of Sciences, 119991 Moscow, Russia; kdem@biochip.ru (E.D.); mafilippova@mail.ru (M.F.); al.vinokurova14@gmail.com (A.V.); sonyagorshkova@gmail.com (S.G.); b.shaskolskiy@biochip.ru (B.S.); grad@biochip.ru (D.G.); 2 State Research Center of Dermatovenerology and Cosmetology of Russian Ministry of Health, Korolenko Street, 3, 107076 Moscow, Russia; alex@cnikvi.ru (A.K.); solomka@cnikvi.ru (V.S.); shagabieva1412@mail.ru (J.S.); dgderyabin@yandex.ru (D.D.)

**Keywords:** *Neisseria gonorrhoeae*, azithromycin resistance, genetic determinants of antimicrobial resistance, G12302 genogroup, NG-MAST, phylogenetic analysis

## Abstract

The goal of this work was to determine the factors affecting the emergence of azithromycin-resistant *Neisseria gonorrhoeae* isolates in Russia, where azithromycin was never recommended for the treatment of gonococcal infections. *Clinical N. gonorrhoeae* isolates collected in 2018–2021 (428 isolates) were analyzed. No azithromycin-resistant isolates were found in 2018–2019, but in 2020–2021, a significant increase in the ratio of azithromycin-resistant isolates was observed: 16.8% and 9.3%, respectively. A hydrogel DNA microarray was developed for the analysis of resistance determinants: mutations in the genes encoding the mtrCDE efflux system and in all four copies of the 23S rRNA gene (position 2611). A majority of the azithromycin-resistant Russian isolates belonged to the NG-MAST G12302 genogroup, and the resistance was associated with the presence of a mosaic structure of the *mtrR* gene promoter region with the −35 delA deletion, an Ala86Thr mutation in the *mtrR* gene, and a mosaic structure of the *mtrD* gene. A comparative phylogenetic study of modern Russian and European *N. gonorrhoeae* populations allowed us to conclude that the emergence of azithromycin resistance in Russia in 2020 was the result of the appearance and spread of European *N. gonorrhoeae* strains belonging to the G12302 genogroup due to possible cross-border transfer.

## 1. Introduction

Gonococcal infection, caused by the Gram-negative bacteria *Neisseria gonorrhoeae*, is a sexually transmitted disease that has a serious negative impact on human sexual and reproductive health. According to WHO data, approximately 82 million new cases of gonococcal infection have recently been reported worldwide each year [1]. In Russia, 10,791 gonorrhoeae cases were recorded in 2021, which was slightly higher than the number in 2020 [2]. A distinctive feature of *N. gonorrhoeae* is that this bacterium can quickly accumulate mutations in its genome, becoming resistant to drugs used for therapy, which creates the risk of gonorrhea becoming an incurable disease [3,4,5].

The main drugs for the treatment of gonococcal infection worldwide are third-generation cephalosporins (ceftriaxone and cefixime), used either as monotherapy or as dual therapy with macrolides (azithromycin) [3,6]. Monitoring the developing resistance to these drugs is extremely relevant. An important tool for tracking the emergence and spread of antibiotic-resistant clones is molecular typing, which aims to identify the most epidemically significant genotypes. The traditional and still widely used molecular typing method is *Neisseria gonorrhoeae* Multi-Antigen Sequence Typing (NG-MAST), based on the determination of the nucleotide sequences of fragments of two hypervariable loci: *porB*, encoding a transmembrane protein of the porin channel, and *tbpB*, encoding the β-subunit of the transferrin-binding protein. The NG-MAST assay allows one to solve two related problems: first, to isolate a significant number of genetic variants (i.e., sequence types) within *N. gonorrhoeae* species and analyze, on this basis, the routes of transmission of gonococcal infection; and second, to identify and control the spread of the most epidemiologically dangerous clones.

An example of the use of this approach is the determination and genetic characterization of NG-MAST 1407, the proportion of which in 2009–2010 was more than 10% of isolates in many European countries [7,8]. Multiple antimicrobial resistance determinants have been found in isolates of this molecular type, including a mosaic type of penicillin-binding protein 2 (PBP2) and a mutation of the Ala501 residue in PBP2 associated with resistance to cephalosporins [7,8,9]. However, a recent paper by Sánchez-Busó L et al. [10] demonstrated a decrease in the G1407 genogroup in the European population: the proportion of G1407 isolates in 2018 was only 2.1%. At the same time, an increase in the number of isolates belonging to the genogroup G12302, which formed relatively recently, was registered. The isolates of this genogroup are associated mainly with decreased susceptibility to macrolides (azithromycin) [10].

Our previous phylogenetic analysis of the NG-MAST types of the Russian isolates collected in 2013–2018 revealed differences between the Russian and European *N. gonorrhoeae* populations [11,12] and allowed us to conclude that the Russian gonococcal population is in some way isolated from the European one and that the evolutionary processes in Russia are highly localized. The majority of the Russian isolates belonged to the NG-MAST types not found in European countries during the time period under study. NG-MAST 807 and 228 were the most common sequence types in Russia, representatives of the G1407 and G12302 genogroups that were detected only sporadically. Against this background, a recent study of Russian *N. gonorrhoeae* isolates collected in 2020–2021 unexpectedly showed a significant proportion of phenotypically azithromycin-resistant isolates: 13.6% [13]. The reasons for the emergence of resistant clones are not fully understood, especially taking into account that azithromycin is not recommended in Russia for the treatment of gonorrhea, although this drug is used to treat other sexually transmitted diseases.

The goal of this work was to establish the cause of the spread of *N. gonorrhoeae* resistance to azithromycin in Russia through a detailed study of *N. gonorrhoeae* isolates obtained in 2018–2021. The objectives of the work included an analysis of the phenotypic susceptibility of isolates to azithromycin, the identification of the genetic determinants of resistance by sequencing and using the original oligonucleotide hydrogel microarray designed in this work, the detection of the most widespread NG-MAST genotypes, and a comparative phylogenetic study of the Russian and European *N. gonorrhoeae* populations.

## 2. Materials and Methods

### 2.1. Collection and Characterization of Russian N. gonorrhoeae Isolates: Antimicrobial Susceptibility Testing

A total of 428 *N. gonorrhoeae* clinical isolates collected by the State Research Center of Dermatovenerology and Cosmetology of the Russian Ministry of Health from nine regions in the Russian Federation (Moscow, Stavropol, Arkhangelsk, Kazan, Cheboksary, Astrakhan, Omsk, Kaluga, and Novosibirsk) were studied (year 2018: 151 isolates, year 2019: 122 isolates, year 2020: 101 isolates, and year 2021: 54 isolates). The participating regions were geographically distributed over the territory of Russia, which allowed the acquisition of representative *N. gonorrhoeae* collections of overall gonorrhea samples during the study period. The samples were obtained by specialized dermatovenerological medical organizations, and each sample came from a separate patient. Cervical specimens of *N. gonorrhoeae* from females and urethral specimens from males were obtained from patients (12 to 64 years of age) with a primary diagnosis (clinically and using the culture method) of symptomatic uncomplicated gonorrhea. The exclusion criteria were as follows: (i) refusal to participate; (ii) negative result on culture or the isolation of non-gonococcal microorganisms; and (iii) the use of antibiotics for the treatment of gonorrhea or other diseases within the last 6 months. The number of refusals was usually low and did not exceed 5%. For each case, the following data were available: sex, age, sexual orientation, information about sexual activity, date the specimen was obtained, specimen site, concurrent STIs (syphilis, trichomoniasis, chlamydia, etc.) diagnosed during that episode, and treatment of STIs in the past. The frequency of co-infection with other STDs did not exceed 10%. At the time of gonococcal infection diagnostics, none of the patients reported the presence of COVID-19 disease, and no laboratory testing for SARS-CoV-2 was performed. The samples were collected, transported, cultured, and stored according to a protocol described previously in Kubanov A et al. [14] as well as in Shaskolskiy B et al. [15].

Although the present study focused on the analysis of *N. gonorrhoeae* resistance to azithromycin, in addition to determining the minimum inhibitory concentration for azithromycin (MIC_azm_), the MIC for ceftriaxone (MIC_cro_) was also measured since ceftriaxone is the drug of choice for gonorrhea treatment in Russia. The measurement of the MICs was performed using serial dilutions in chocolate agar. Each strain was characterized in accordance with the EUCAST criteria [16]:

Azithromycin: S—susceptible (MIC_azm_ ≤ 1.0 mg/L), R—resistant (MIC_azm_ > 1.0 mg/L);

Ceftriaxone: S—susceptible (MIC_cro_) ≤ 0.125 mg/L), R—resistant (MIC_cro_ > 0.125 mg/L).

### 2.2. Detection of Mutations Associated with Azithromycin Resistance Using an Oligonucleotide Hydrogel Microarray

The molecular determinants associated with azithromycin resistance in the Russian clinical *N. gonorrhoeae* isolates were determined using hydrogel-based DNA microarrays designed in this work. The microarrays were manufactured using a copolymerization immobilization method according to a procedure previously developed at the Engelhardt Institute of Molecular Biology, Russian Academy of Sciences [17]. The microarray was a plastic substrate with deposited hydrogel elements (droplets), and each element contained an immobilized oligonucleotide probe.

**Figure 1:**
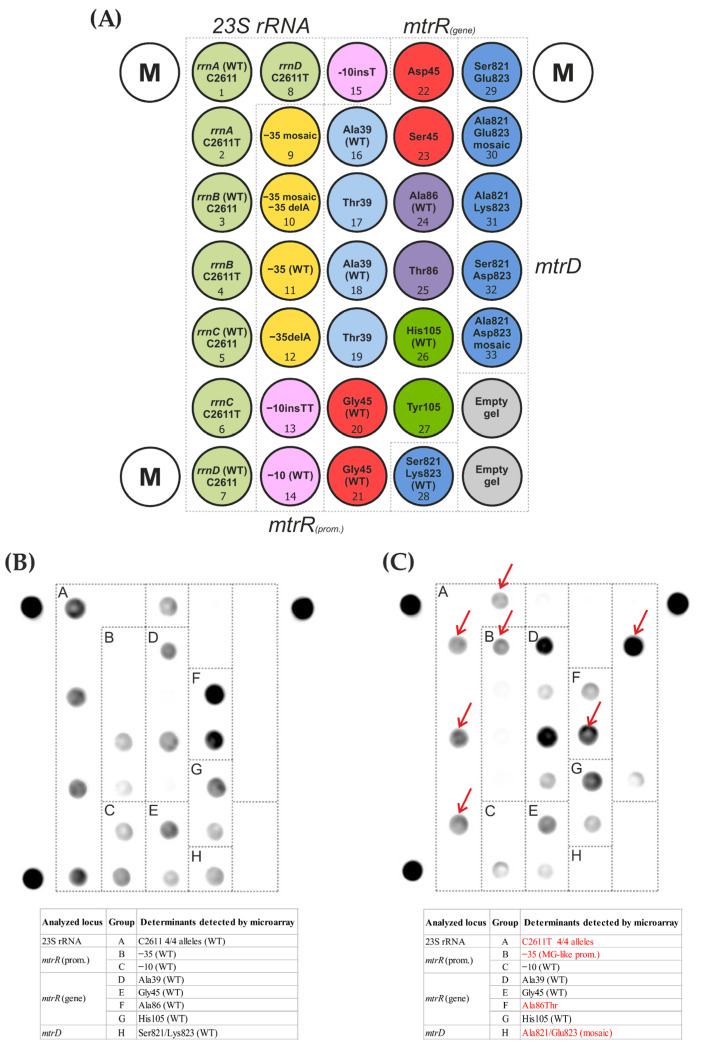
DNA hydrogel microarray for detecting mutations in the *N. gonorrhoeae* 23S rRNA genes *mtrR* (coding and promoter regions) and *mtrD* associated with resistance to azithromycin. (**A**). Configuration of the microarray. The microarray contained 33 elements with immobilized oligonucleotide probes, 2 elements without probes (empty gels), and 3 marker elements (M) for the proper image acquisition and processing of the fluorescent signals. The microarray elements are drawn as circles inside, where the analyzed mutations are indicated. The number inside each circle corresponds to the number of immobilized probes (the sequences of the probes are provided in Supplementary File S1). The groups of elements (8 groups) for the analysis of the mutations in the different loci are represented by different colors. (**B**,**C**). Fluorescence hybridization patterns of the microarrays after the analysis of genomic DNA obtained from different *N. gonorrhoeae* isolates. The groups of elements are designated by dashed lines. The elements with detected mutations are noted with red arrows. (**B**) A wild-type isolate (wt) carrying no mutations in the 23S rRNA, *mtrR*, or *mtrD* genes. (**C**) Isolate carrying the C2611T mutation in all four alleles of the 23S rRNA gene, the mosaic (Meningitidis-like) *mtrR* promoter, the Ala86Thr mutation in the *mtrR* gene, and the Ser821Ala and Lys823Glu mutations in the mosaic *mtrD* gene.

The configuration of the microarray containing oligonucleotide probes for the identification of mutations in the 23S rRNA gene (position 2611), the *mtrR* gene (codons 39, 45, 86, and 105), the *mtrD* gene (codons 821 and 823), and the promoter region of the *mtrR* gene (positions −10 and −35) is shown in Figure 1A. The microarray contained 33 hydrogel elements with immobilized oligonucleotide probes, three marker elements (M) for image acquisition with processing software, and two empty hydrogel elements for signal normalization. The microarray elements were distributed into eight groups according to the locus at which the presence or absence of the mutation was analyzed (highlighted by colors in Figure 1), for example, the group of elements 1–8 for the identification of the C2611T mutation in 23S RNA and the group of elements 9–12 for the identification of the deletion in position −35 of the promoter region of the *mtrR* gene. When selecting the nucleotide sequences of the probes for the analysis of each molecular determinant, the presence of the different types of the *mtrR* and *mtrD* alleles was considered: wild-type, nonmosaic, or mosaic. The selection of the probes for the identification of mutations in the 23S rRNA genes was performed by taking into account all four copies of the *rrn* operon, which allowed for the presence or absence of mutations in each individual copy of the 23S rRNA gene to be recorded.

The assay procedure included multiplex PCR amplification to obtain predominantly single-stranded fluorescence-labeled DNA fragments, hybridization of the DNA fragments on microarrays, and fluorescence image acquisition and analysis. The sequences of the immobilized probes and primers used for the multiplex PCR and the protocols used for the PCR and microarray hybridization are provided in Supplementary File S1. The acquisition of the fluorescence images and the measurement of the signal intensities were performed using a proprietary microarray scanner equipped with the original software (Biochip-IMB, Ltd., Moscow, Russia). The fluorescence signals were analyzed separately within each group of elements (Figure 1) to determine the maximum positive signal within the group, indicating the presence or absence of a mutation in the analyzed locus. Examples of the fluorescence images of the microarrays after the analysis (i.e., fluorescence hybridization patterns) are shown in Figure 1B,C.

### 2.3. Target Sequencing of N. gonorrhoeae DNA

The results obtained using DNA microarrays were confirmed by Sanger sequencing using a 3730xl Genetic Analyzer (Applied Biosystems, Foster City, CA, USA). The mutations in the positions A2058, A2059, and C2611 of the 23S rRNA gene (four copies) and in the *mtrR* gene were determined using a previously described method [18]; the *mtrD* gene was sequenced using the primers indicated in Kandinov I et al. [13]. The presence of *ermA/B/C/F* methyltransferase genes and the *mefA* gene in *N. gonorrhoeae* was checked as described in Cousin S et al. [19].

### 2.4. NG-MAST Typing of the Russian N. gonorrhoeae Isolates and Definition of the Genogroups

The molecular typing of *N. gonorrhoeae* isolates was carried out using the standard NG-MAST protocol. The variable internal regions of the *porB* and *tbpB* genes were amplified by PCR, and the resulting products were purified and sequenced. The allele numbers for the *porB* and *tbpB* sequences and sequence types were assigned according to the NG MAST v2.0 database (https://pubmlst.org, accessed on 15 December 2022).

A phylogenetically related genogroup was defined as described in [8] as the set of the *porB* and *tbpB* alleles (variable internal regions) for which the concatenated sequence of both alleles (880 bp) displayed a 99.4% (875 bp) similarity to the concatenated sequence of both alleles of the main ST in the genogroup. The similarity of alleles was evaluated in MEGA X.

### 2.5. Analysis of the Collection of the Russian and European Samples

To compare the Russian and European isolates, we used the results of a 2018 study of 2375 *N. gonorrhoeae* isolates from 26 European countries (Euro-GASP 2018 genomic survey, published in Sánchez-Busó L et al. [10]. The collection of isolates is available in the Pathogenwatch database: https://pathogen.watch/collection/eurogasp2018, accessed on 15 December 2022). A total of 1578 isolates with established MICs to azithromycin, NG-MAST types, *porB*, *tbpB*, *mtrR*, *mtrD*, and 23S rRNA allele types were selected for analysis. The isolates with unspecified MICs as well as novel and unidentified alleles were not considered.

For a comparative analysis of the NG-MAST types of EU countries and Russia, the isolates obtained in Russia were assigned to the European genogroups described in [10].

### 2.6. Construction of Phylogenetic Trees

The *porB* and *tbpB* sequences were concatenated and imported into the alignment tool Bio-Edit (Ibis biosciences, Carlsbad, CA, USA). A maximum likelihood phylogenetic tree was generated using RaxML software, version 8.2.4 (https://usegalaxy.eu/, accessed on 26 December 2022), with 1000 rapid bootstrap inferences. A circular cladogram was constructed using NG-MAST data for all 428 N. gonorrhoeae isolates obtained in Russia. A phylogram was constructed for the NG-MAST types of the European and Russian isolates that form genogroups (a total of 182 sequencing types and 21 genogroups). The obtained trees were visualized in FigTree v1.4.4 software (http://tree.bio.ed.ac.uk/, accessed on 26 December 2022).

## 3. Results

### 3.1. Azithromycin Susceptibility and Genetic Resistance Determinants of Isolates Collected in Russia in 2018–2021

Testing of the 428 clinical isolates for susceptibility to azithromycin did not reveal resistant isolates in the 2018 (n = 151) and 2019 (n = 122) samples, whereas in the 2020–2021 samples, 17 isolates out of 101 (16.8%) and 5 isolates out of 54 (9.3%) had a MIC_azm_ > 1 mg/L, which, according to the EUCAST criteria, rated them as resistant. All characteristics of the studied isolates (i.e., MICs, detected mutations, and NG-MAST types) are provided in Supplementary Materials, Table S1.

The detected mutations associated with azithromycin resistance are shown in Table 1. The C2611T mutations in the 23S rRNA gene that determine “specific” resistance to azithromycin were found in only two resistant isolates in the 2020 isolate sample. At the same time, the mutations associated with the antibiotic efflux system, which provide resistance to a variety of drugs, were quite numerous and included substitutions in the *mtrR* and *mtrD* genes of the MtrCDE efflux pump: Ala39Thr, Gly45Asp/Ser, Ala86Thr, and His105Tyr substitutions in the *mtrR* gene; −35 delA and −10 insT (this mutation was found in only one isolate) in the promoter region of the *mtrR* gene; and the presence of mosaic alleles of the *mtrR* (promoter region) and *mtrD* (Ala821/Glu823 mosaic) genes.

As can be seen from Table 1, there is a good correspondence between phenotypic resistance to azithromycin and the accumulation of the indicated mutations. All of the 2018–2019 azithromycin-susceptible isolates generally lacked *mtrR* and *mtrD* mosaic alleles. Some of them had substitutions in nonmosaic alleles of the *mtrR* and *mtrD* genes; however, such mutations did not lead to resistance. In 2020–2021, azithromycin-resistant isolates were found, particularly isolates with mosaic alleles of these genes. In 2020, 14% and in 2021, 22.2% of the isolates carried mosaic alleles of the *mtrR* gene (promoter region): −35 mosaic and −35 mosaic/delA; in addition, 18% and 33% of the isolates, respectively, carried mosaic alleles of the *mtrD* gene. As a rule, resistant isolates possessed mosaic alleles simultaneously in the *mtrR* and *mtrD* genes.

No isolates with A2058G/A2059G substitutions in 23S RNA, which can lead to a MIC_azm_ > 256 mg/L [20], were found in the samples under study. No *erm* methyltransferase genes or the *mefA* efflux pump gene, which, as noted in the literature, are rarely found in the population [19,21], were detected.

### 3.2. Comparison of the NG-MAST Types in the Russian and European N. gonorrhoeae Populations

According to the NG-MAST typing results, 428 Russian isolates collected in 2018–2021 belonged to 161 NG-MAST types, including 69 NG-MAST types that were also found in Europe and 92 unique Russian NG-MAST types not found in Europe. The European types included 244 isolates (57.0%) (i.e., more than half of the isolates in the studied sample), and 184 isolates (43.0%) belonged exclusively to the Russian population.

The Russian isolates under study were allocated to the European genogroups defined on the basis of the results of the study of *N. gonorrhoeae* isolates obtained in 2018 in 26 European countries [10] (Table 2). For example, the *N. gonorrhoeae* isolates with NG-MAST 807, NG-MAST 228, etc., collected in Russia and differing from NG-MAST 10800 by no more than five nucleotides in a combination of *porB* and *tbpB* alleles, were assigned to the European genogroup G10800. Some NG-MASTs were found only in the Russian population, and they were grouped into the Russian genogroups that were named after the most frequently found STs in the group, e.g., G6226_(RUS)_ was named after NG-MAST 6226.

The analysis showed the presence of a large number of “overlapping” genogroups, i.e., genogroups in which both European and Russian NG-MAST types were present, such as G10800, G1993, G12302, G387, and G1407. It should be noted, however, that the same genogroup in Europe and in Russia included isolates of different sequence types; for example, G387 in Russia mainly consisted of isolates of NG-MAST 9486, and the European G387 included isolates of NG-MAST 387 and 5743. Three genogroups, G6226, G5042, and G14942, which include sequencing types found only in Russia, and three European genogroups, G11461, G14994, and G14769, consisting only of European NG-MAST types, can be distinguished. However, despite the earlier demonstrated genetic remoteness of the Russian and European populations, the gradual increase in the number of “overlapping” genogroups in Russia and Europe may indicate migrations between populations.

**Figure 2:**
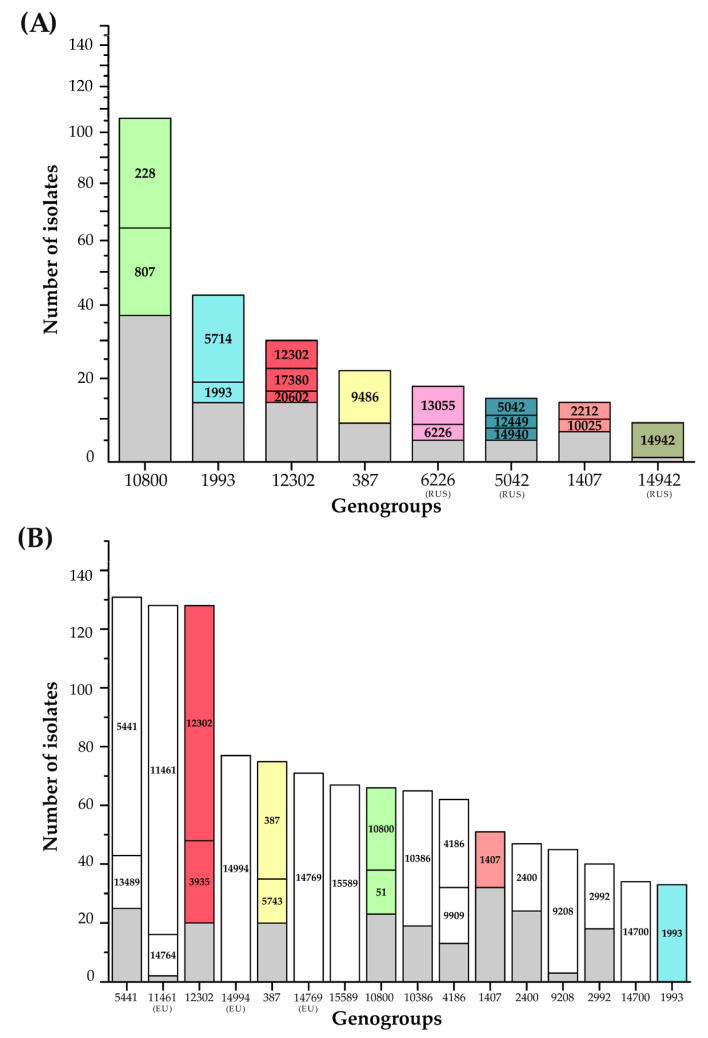
Comparison of the genogroups for the Russian (**A**) and European (**B**) N. gonorrhoeae populations. Genogroups with a (RUS) index include isolates of the Russian NG-MAST types not found in European countries; those with an (EU) index include isolates of the sequence types found only in Europe. The numbers inside the columns show the main NG-MAST types of the genogroup; NG-MAST types with the highest number of isolates are highlighted by colors, with gray indicating the remaining types. The colors representing the genogroups in Figure 2, Figure 3 and Figure 4 are the same.

We performed a comparative analysis of the distribution of the isolates by genogroup for the Russian and European *N. gonorrhoeae* populations (Figure 2). The G10800 genogroup was the most abundant in Russia, comprising 103 isolates (24% of the studied sample). It included the isolates belonging to NG-MAST types 228 and 807 (35 and 34 isolates, respectively), which for many years were the most abundant sequence types in the Russian population [11,22]. Interestingly, no isolates of NG-MAST 10800, which defines the nomenclature of this genogroup in Europe, were found in Russia. Second place belonged to genogroup G1993, which also had a wide distribution in Russia in 2013–2018. Genogroup G12302 (30 isolates, 7%) was in third place. G12302 included only two isolates in the years 2018–2019, while there were 19 isolates collected in 2020 and 9 isolates collected in 2021. Most of the Russian azithromycin-resistant isolates (13 out of 17) belonged to the G12302 genogroup. Genogroup G12302 is currently one of the most common in Europe (see Figure 2B) and is also a rapidly growing genogroup associated with azithromycin resistance [10].

As noted by Sánchez-Busó L et al. [10], in parallel with the growth of genogroup G12302 in Europe, the epidemiologically dangerous genogroup G1407, associated with cephalosporin resistance, has been gradually decreasing since 2012–2013. The only ceftriaxone-resistant isolate with a MIC_cro_ = 0.5 mg/L from Spain belonged to genogroup G3435 rather than G1407. However, in the Russian population of *N. gonorrhoeae*, the G1407 genogroup continued to increase and expand; for example, of the six isolates of NG-MAST 3149, 5622, and 10025 obtained in 2018/19, eight more that were isolated and found to belong to ST 2212, 4706, 20278, 20283, and 20285 were added in 2020/21. Among the Russian isolates studied in this work, two isolates with decreased susceptibility to ceftriaxone with a MIC_cro_ = 0.12 mg/L were found: one isolate of NG-MAST 5622 (year 2018) and another of NG-MAST 10025 (year 2019), and these isolates belonged to the particular genogroup G1407. Both isolates were susceptible to azithromycin (see Supplementary Materials, Table S1).

### 3.3. Phylogenetic Study of the Russian Isolates

**Figure 3:**
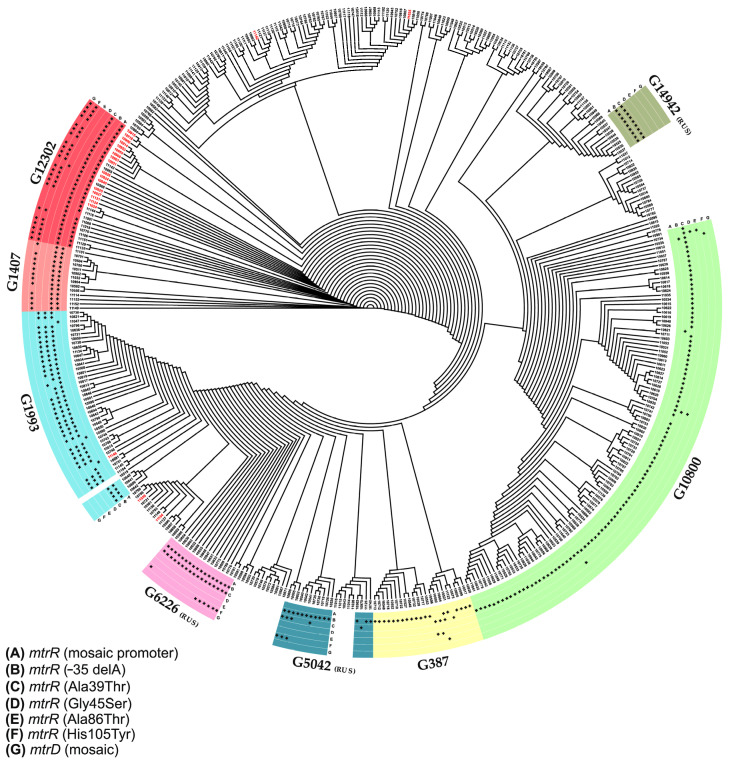
Maximum likelihood phylogenetic tree for the Russian isolates based on the NG-MAST typing data. The numbers on the perimeter of the circle correspond to the sample code of the isolate, and the sample codes of azithromycin-resistant isolates are marked in red. The main genogroups (G10800, G387, etc.) are marked by different colors. Within the genogroups, the detected mutations (A–G) associated with azithromycin resistance are marked by ‘plus’ signs. Thus, our results on the affiliation of most azithromycin-resistant Russian isolates of genogroup G12302 and on the identified resistance determinants are in good agreement with the results of the analysis of the European isolates, where resistance to azithromycin in the new European spreading genogroup G12302 is also associated with the presence of mosaic structures of the *mtrR* (promoter region) and *mtrD* genes. These results suggest that the emergence of azithromycin-resistant *N. gonorrhoeae* isolates belonging to genogroup G12302 in Russia in 2020–2021 is not a consequence of the evolution of the Russian clones but most likely can be explained by the migration of *N. gonorrhoeae* strains from European countries.

A phylogenetic tree constructed from the NG-MAST typing data for all 428 Russian isolates is shown in Figure 3. For a detailed analysis of azithromycin resistance, the resistant isolates within genogroups and detected mutations associated with resistance to this drug are marked in the tree. The tree clearly demonstrates a large phylogenetic distance among isolates of the G10800 genogroup, which included approximately one-quarter of all isolates and, in particular, the most frequent isolates of Russia’s NG-MAST types 228 and 870. On the other hand, one can see the phylogenetic proximity of the isolates assigned to the most common European genogroups G1407 and G12302, which are also quite distant from the other identified genogroups.

Numerous point mutations in the antibiotic efflux system associated with resistance were found in the isolates that retained phenotypic susceptibility to azithromycin; for example, almost all isolates of genogroup G10800 carried an Ala86Thr mutation in the *mtrR* gene, and isolates of genogroup G5042 carried a His105Tyr mutation in the *mtrR* gene; isolates of genogroups G6226 and G14942 carried Ala86Thr and His105Tyr mutations in the *mtrR* gene; isolates of genogroup G1407 had −35 delA mutations in the promoter region of the *mtrR* gene and Ala86Thr and His105Tyr mutations in the *mtrR* gene; and isolates of genogroup G1993 had Ala39Thr, Gly45Ser, and Ala86Thr mutations in the *mtrR* gene. However, isolates of all of the named genogroups did not have the mosaic structure of the *mtrR* and *mtrD* genes, and even the simultaneous presence of three mutations in the nonmosaic *mtrR* and *mtrD* genes did not lead to resistance to azithromycin.

In the studied sample, most of the azithromycin-resistant isolates (17 of 22) belonged to the G12302 genogroup and had a mosaic structure of the *mtrR* gene promoter region with the −35 delA deletion and Ala86Thr mutation and a mosaic structure of the *mtrD* gene with the Ser821Ala and Lys823Gly substitutions. One of the five resistant isolates not belonging to genogroup G12302 (sample code 11039, NG-MAST 16169) had a C2611T mutation in the 23S rRNA gene (4/4 alleles); another isolate (sample code 11142, NG-MAST 4550) had a mosaic structure with a −35 delA deletion; and three isolates from genogroups G1993, G5624, and G19572_(RUS)_ had no mosaic alleles, and only Ala39Thr, Ala86Thr, and His105Tyr substitutions in the *mtrR* gene were detected (see Supplementary Materials, Table S1).

## 4. Discussion

The drug of choice for the therapy of gonococcal infection in Russia is ceftriaxone, and, unlike in European countries, azithromycin is not recommended in Russia for the therapy of gonococcal infection, although this drug is used for the treatment of other sexually transmitted diseases. *N. gonorrhoeae* isolates phenotypically resistant to azithromycin were found in Russia in 2007–2015 [14], but their frequency of occurrence was sporadic. In the present work, 428 *N. gonorrhoeae* isolates obtained in Russia in 2018–2021 were analyzed. No azithromycin-resistant isolates were found in the Russian *N. gonorrhoeae* population in 2018/2019, but in 2020/2021, a significant proportion of resistant isolates was registered: 16.8% in 2020 and 9.3% in 2021, which is well above the threshold value of 5% recommended by the WHO for the inclusion/exclusion of the drug from therapy regimens.

To identify the genetic determinants associated with *N. gonorrhoeae* resistance to azithromycin, a specialized DNA microarray was developed in this work. A distinctive feature of the microarray is the ability to analyze mutations in all four copies of the 23S rRNA operon (position 2611). This is important because the level of resistance of *N. gonorrhoeae* to azithromycin depends on the number of mutant alleles of the 23S rRNA; high resistance is observed in isolates containing 3–4 mutant alleles, and insignificant resistance is observed in isolates with one copy of the mutant allele [23].

The analysis of the genetic determinants showed that the mosaic alleles of the *mtrR* (promoter region) and *mtrD* genes were the main determinants of the resistance of *N. gonorrhoeae* to azithromycin, and the number of isolates carrying mutations in the 23S rRNA gene decreased both in Europe [10] and in Russia. Mosaic alleles are assumed to have formed as a result of horizontal gene transfer from other *Neisseria* species [24,25]. Almost all of the phenotypically azithromycin-susceptible Russian isolates did not carry mosaic alleles of the *mtrR* and *mtrD* genes.

A significant proportion (43.0%) of the analyzed isolates of the Russian *N. gonorrhoeae* population belonged to sequence types found exclusively in Russia; 57.0% of the isolates belonged to sequence types that are widespread in European countries. It is interesting to note that the Russian *N. gonorrhoeae* population “lags behind” the European population. Thus, genogroup G1407, considered epidemiologically dangerous in the world because of the presence of multiresistant isolates, has been decreasing in Europe since 2012–2013 [10] due to the introduction of dual therapy for gonococcal infection (azithromycin + ceftriaxone) in 2012 [26]. In Russia, however, the G1407 genogroup continued to increase in 2018–2021, with six G1407 genogroup isolates detected in 2013–2017, six G1407 isolates in 2018/19, and eight G1407 isolates in 2020/21. It can be assumed that the growth of genogroup G1407 in Russia may be related to the use of mainly ceftriaxone for the therapy of gonococcal infection (no dual therapy including azithromycin). No ceftriaxone-resistant isolates have been identified in Russia so far, but two isolates of genogroup G1407 had a MIC_cro_ = 0.12 mg/L, i.e., at the threshold value between resistant/susceptible isolates according to the EUCAST criterion.

**Figure 4:**
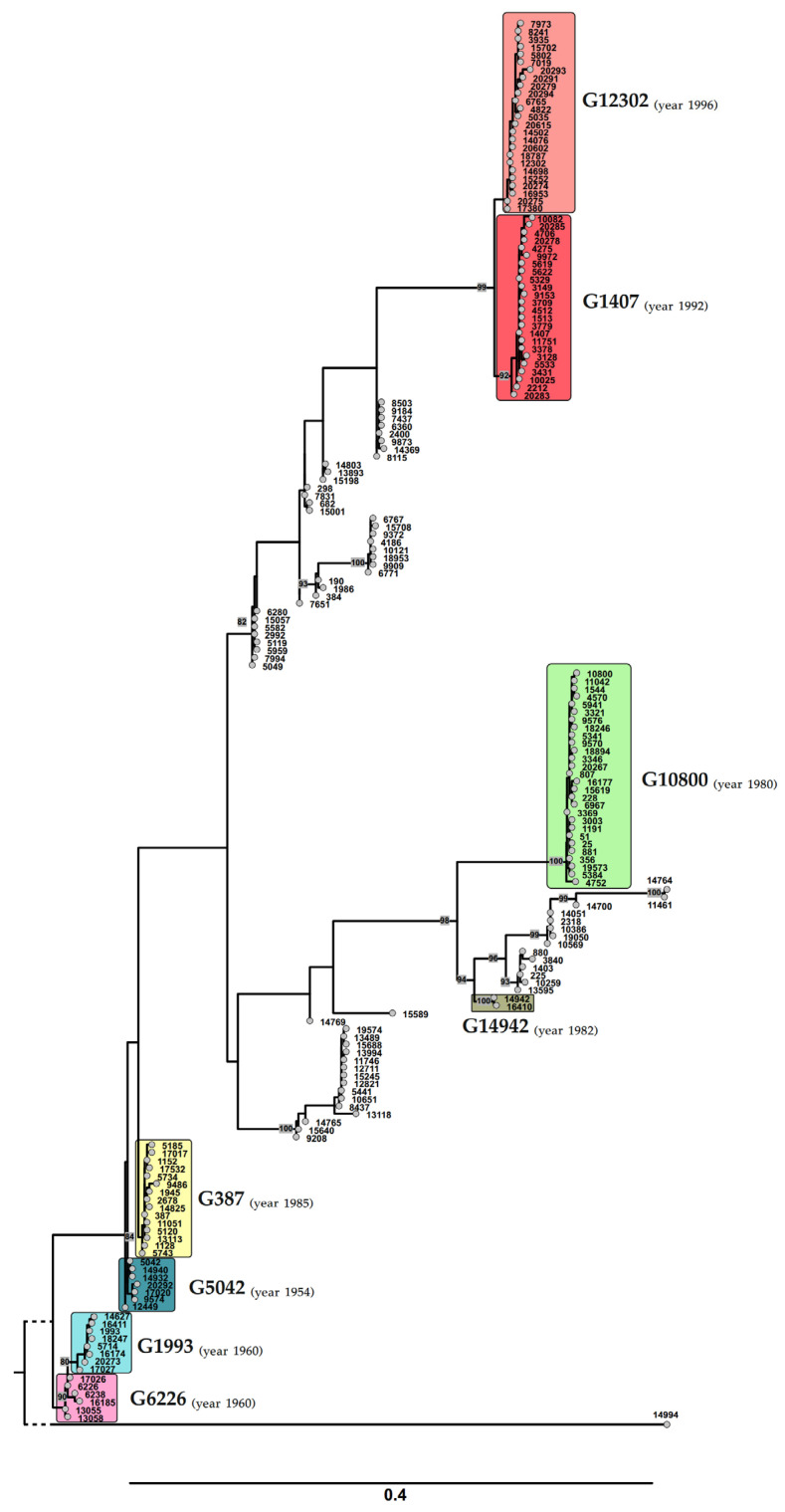
Phylogram of the NG-MAST types of the European and Russian isolates forming the main genogroups. The numbers indicate the NG-MAST types. The year of detection of the first NG-MAST in a genogroup according to the PubMLST database is also indicated.

Evolutionary distances between the sequences and genogroups of the Russian and European isolates can be seen in the phylogram, which also shows the year of detection of the first isolate of a particular genogroup according to the PubMLST database (Figure 4). The G6226_(RUS)_ and G1993 genogroups are phylogenetically close, but G6226_(RUS)_ includes only NG-MAST types found in Russia, whereas the G1993 genogroup includes both the Russian and European NG-MAST types. Thus, genogroup G6226, first detected in the world in 1960, has remained only in Russia and is gradually being replaced in Russia by genogroup G1993, which was revealed at the same time. The G5042_(RUS)_ and G387 genogroups are phylogenetically close. The G5042_(RUS)_ genogroup, first revealed in 1954, includes only Russian NG-MAST types, whereas the G387 genogroup (year 1985) includes both Russian and European NG-MAST types. Apparently, the G5042_(RUS)_ genogroup, which is retained only in Russia, is gradually being replaced in Russia by the European G387 genogroup.

Figure 4 demonstrates that the G1407 and G12302 genogroups, which include both Russian and European NG-MAST types, most likely descended from a single ancestor. NG-MAST 1407 and NG-MAST 12302 have the same *porB* allele (allele number 908) and differ only in the *tbpB* allele (allele numbers 110 and 267, respectively, according to the pubMLST database).

The emergence of azithromycin-resistant *N. gonorrhoeae* isolates coincided with the 2020–2021 COVID-19 pandemic. Since azithromycin was indeed administered to Russian COVID-19 patients at the early stages of the pandemic, it can be assumed that its use may have influenced the formation of an azithromycin-resistant clone of *N. gonorrhoeae* isolates, leading to its origin from traditional Russian gonococcal genogroups. However, our phylogenetic study clearly showed that the emerging genogroup is far from the Russian endemic G10800 genogroup and corresponds to the European G12302 genogroup.

At present, a simultaneous decrease in the G1407 genogroup and an increase in the G12302 genogroup (which appeared in 1996) have been observed in Europe. Correspondingly, the resistance of *N. gonorrhoeae* to ceftriaxone and cefixime associated with the G1407 genogroup has been reduced, but the resistance to azithromycin associated with the G12302 genogroup has increased. According to a study by Sánchez-Busó et al. [10], azithromycin resistance in the European population has been increasing in recent years, precisely because of the emergence and spread of the G12302 genogroup, which included 5.6% of the European isolates from 24 countries in 2018. NG-MAST 12302/MLST 9363 appeared to be the most frequent type in Austria in 2016–2020, and 71% of the isolates with this ST were resistant to azithromycin [27]. The widespread distribution of NG-MAST 12302/MLST 9363 has also been reported in the USA [28] and Canada [29] and has been associated with azithromycin resistance. The resistance determinants involved the presence of the mosaic structures of the *mtrR* (promoter region) and *mtrD* genes of the MtrCDE efflux pump. As shown in this work, a majority of the azithromycin-resistant Russian isolates belonged to the G12302 genogroup and had a mosaic structure of the *mtrR* gene promoter region with a −35 delA deletion, an Ala86Thr substitution, and a mosaic structure of the *mtrD* gene. Thus, it can be concluded that the resistance of *N. gonorrhoeae* to azithromycin that emerged in 2020 is associated with the appearance and spread in Russia of European G12302 genogroup strains due to the possible occurrence of cross-border transfer.

The observed growth of genogroup G12302 and the increasing resistance to azithromycin may jeopardize the dual therapy of gonococcal infection with ceftriaxone and azithromycin recommended in Europe. As for the Russian Federation, where two epidemiologically dangerous genogroups, G1407 and G12302, associated with cephalosporin and macrolide resistance, respectively, are simultaneously increasing, one should be wary of the growth of failures in gonococcal therapy.

## 5. Conclusions

Despite the fact that azithromycin is not recommended in Russia for the treatment of gonococcal infection, an increase in the resistance of *N. gonorrhoeae* to this drug was registered in 2020–2021: 16.8% in 2020 and 9.3% in 2021. The majority of the azithromycin-resistant isolates belonged to the G12302 group and had a mosaic structure of the promoter region of the *mtrR* gene with a −35 delA deletion, an Ala86Thr mutation in the coding region of the *mtrR* gene, and a mosaic structure of the *mtrD* gene.

The azithromycin resistance in the Russian *N. gonorrhoeae* population that emerged in 2020 is associated with the spread of European *N. gonorrhoeae* strains of the G12302 genogroup due to the possible occurrence of cross-border transfer.

## Figures and Tables

**Table 1:** Mutations associated with azithromycin resistance in *N. gonorrhoeae* isolates collected in Russia in 2018–2021.

Analyzed Locus	Detected Determinants	Proportion of Isolates			
		Year 2018	Year 2019	Year 2020	Year 2021
23S rRNA	C2611 (4/4 alleles)	100%	100%	98%	100%
	C2611T (4/4 alleles)	-	-	2%	-
*mtrR* (promoter)	−35 (wt)	82.8%	90%	76%	64.8%
	−35 delA	16.5%	10%	10%	13%
	−35 mosaic	-	-	-	3.7%
	−35 mosaic/delA	0.7%	-	14%	18.5%
	−10 (wt)	99.3%	100%	100%	100%
	−10 insT	0.7%	-	-	-
*mtrR* (coding region)	Ala39 (wt)	83%	78%	80%	55.5%
	Thr39	17%	22%	20%	44.5%
	Gly45 (wt)	83%	79%	83%	90.8%
	Asp45	8%	9%	8%	3.7%
	Ser45	9%	12%	9%	5.5%
	Ala86 (wt)	24.5%	24%	10%	13%
	Thr86	75.5%	76%	90%	87%
	His105 (wt)	99.3%	98.4%	82%	67%
	Tyr105	0.7%	1.6%	18%	33%
*mtrD*	Ser821/Lys823 (wt)	99.3%	98.4%	82%	67%
	Ala821/Glu823 (mosaic)	0.7%	1.6%	18%	33%
Total number of isolates for each year		151	122	101	54
Number of phenotypically azithromycin-resistant isolates		0	0	17 (16.8%)	5 (9.3%)

**Table 2:** Distribution of the Russian isolates (428 isolates) by genogroups according to the genogroups detected in European countries in 2018 [10]. Genogroups with the _(RUS)_ index include isolates of the NG-MAST types found in Russia but not in European countries.

Geno- Group	Number of Isolates (%)	NG-MAST (Number of Isolates)	Azithromycin Susceptibility	Median MIC_azm_, mg/L
G10800	103 (24.0%)	228 (35); 807 (34); 1544 (3); 3321 (2); 3369 (1); 4570 (2); 5941 (5); 6967 (1); 9570 (6); 9576 (3); 15619 (1); 16177 (1); 18246 (2); 18894 (5); 19573 (1); 20267 (1)	S (all isolates)	0.12
G1993	43 (10.0%)	1993 (23); 5714 (6); 14627 (3); 16174 (2); 16411 (2); 17027 (4); 18247 (1); 20273 (2)	S (42 isolates) R (1 isolate)	0.12
G12302	30 (7.0%)	6765 (1); 12302 (7); 14502 (2); 16953 (2); 17380 (6); 18787 (2); 20274 (1); 20275 (1); 20279 (1); 20291 (1); 20293 (1) 20294 (1); 20602 (3); 20615 (1)	S (13 isolates) R (17 isolates)	2.0
G387	22 (5.1%)	1152 (2); 5185 (1); 5734 (2); 9486 (13); 14825 (1); 17017 (2); 17532 (1)	S (all isolates)	0.06
G1407	14 (3.3%)	2212 (3); 3149 (1); 4706 (2); 5622 (1); 10025 (4); 20278 (1); 20283 (1); 20285 (1)	S (all isolates)	0.25
G14020	9 (2.1%)	14020 (9)	S (all isolates)	0.06
G12542	3 (0.7%)	12542 (3)	S (all isolates)	-
G5441	2 (0.5%)	13994 (1); 19574 (1)	S (all isolates)	-
G799	2 (0.5%)	19588 (2)	S (all isolates)	-
G4186	2 (0.5%)	18953 (2)	S (all isolates)	-
G5624	2 (0.5%)	5624 (2)	S (1 isolate), R (1 isolate)	-
G9918	2 (0.5%)	20614 (2)	S (all isolates)	-
G10386	2 (0.5%)	19050 (2)	S (all isolates)	-
G14700	2 (0.5%)	14700 (2)	S (all isolates)	-
G15589	2 (0.5%)	15589 (2)	S (all isolates)	-
G5	1 (0.2%)	21 (1)	S	-
G758	1 (0.2%)	20280 (1)	S	-
G3785	1 (0.2%)	18131 (1)	S	-
G2400	1 (0.2%)	9184 (1)	S	-
G12818	1 (0.2%)	18245 (1)	S	-
G6226_(RUS)_	18 (4.2%)	6226 (9); 13055 (4); 13058 (2); 6238 (1); 16185 (1); 17026 (1)	S (all isolates)	0.12
G5042_(RUS)_	15 (3.5%)	5042 (4); 9574 (1); 12449 (3); 14940 (3); 17020 (2); 14932 (1); 20292 (1)	S (all isolates)	0.09
G14942_(RUS)_	9 (2.1%)	14942 (8); 16410 (1)	S (all isolates)	0.12
G19572_(RUS)_	7 (1.6%)	19572 (6), 19576 (1)	S (6 isolates), R (1 isolate)	0.12
Un-grouped	133 (33.2%)	-	S (131 isolates) R (2 isolates)	0.12

## Data Availability

Sequences of the new *mtrR* alleles found in the Russian *N. gonorrhoeae* isolates in 2020–2021 were added to the NG-STAR database under the following accession numbers: alleles 525, 526, 528, 529, and 530. A new NG-MAST ST 21289 was added to the PubMLST database (https://pubmlst.org/bigsdb?page=profileInfo&db=pubmlst_neisseria_seqdef&scheme_id=71&profile_id=21289, accessed on 12 April 2023).

## Supplementary Materials

The following supporting information can be downloaded at: https://www.mdpi.com/article/10.3390/microorganisms11051226/s1, Supplementary File S1: Identification of mutations in *N. gonorrhoeae* genes associated with resistance to azithromycin using DNA microarrays; Table S1: Characteristics of *N. gonorrhoeae* isolates collected in the Russian Federation in 2018–2021.
