# Supplementary material for: Emergence of Azithromycin-Resistant Neisseria gonorrhoeae Isolates Belonging to the NG-MAST Genogroup 12302 in Russia
Source: Microorganisms. 2023 May 6;11(5):1226. doi: 10.3390/microorganisms11051226 (PMC10222849; doi:10.3390/microorganisms11051226)
Supplement: Supplementary file 1 [file microorganisms-11-01226-s001.zip › Supplementary File S1.pdf]

Supplementary File S1: Identification of mutations in *N. gonorrhoeae* genes associated with the resistance to azithromycin using DNA microarrays

Microarray layout:

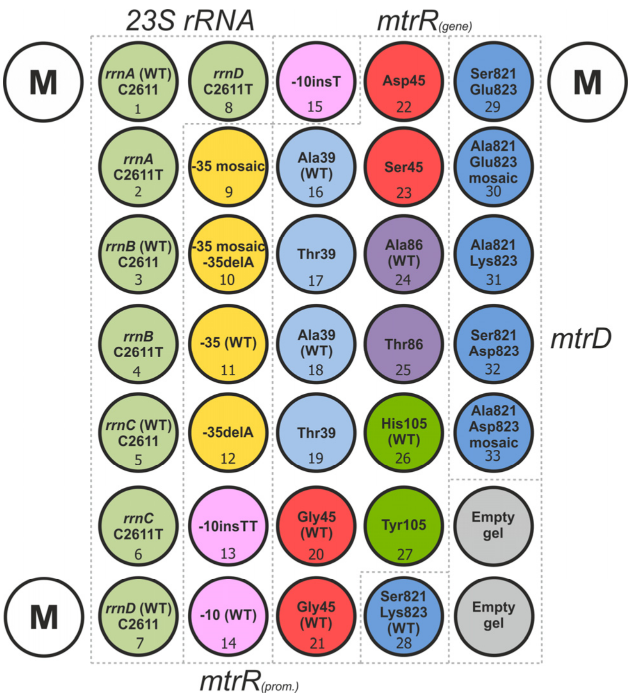

Immobilized probes:

	Group	Locus	Probe sequence, 5'-3'	Analysed mutation
1	A	23S rRNA	CGACCGGAGATAGGGACCAAA	*rrnA* (wt) C2611
2			CGACCGGAGATAGAGACCAAA	*rrnA* C2611T
3			GCAGTGCTGATAGGGACCAAA	*rrnB* (wt) C2611
4			GCAGTGCTGATAGAGACCAAA	*rrnB* C2611T
5			CCTCTCAAGATAGGGACCAAA	*rrnC* (wt) C2611
6			CCTCTCAAGATAGAGACCAAA	*rrnC* C2611T
7			CTACAGCGGATAGGGACCAAA	*rrnD* (wt) C2611
8			CTACAGCGGATAGAGACCAAA	*rrnD* C2611T
9	B	*mtrR* promoter	ATTGCACGGATACAAAGTCTTT	−35 mosaic
10			ATTGCACGGATCAAAGTCTTT	−35 mosaic/delA
11			ATTGCACGGATAAAAAGTCTTT	−35 (wt)
12			ATTGCACGGATAAAAGTCTTT	−35delA
13	C		AAAGTCTTTTTTTTATAATCCGCC	−10insTT
14			AAAGTCTTTTTTATAATCCGCC	−10 (wt)
15			AAAGTCTTTTTTTATAATCCGCCC	−10insT
16	D	*mtrR* gene coding region	TCGCCCAAGCCGCCGGC	Ala39 (wt)
17			TCGCCCAAACCGCCGGC	Thr39
18			ATCGCCCAAGCCGCCGG	Ala39 (wt)
19			ATCGCCCAAACCGCCGG	Thr39
20	E		AACGCGCGGCGCGCTCT	Gly45 (wt)

21		*mtrR* gene coding region	AACGCGCGGCACGCTCT	Gly45 (wt)
22			AACGCGCGACGCGCTCT	Asp45
23			AACGCGCAGCGCGCTCT	Ser45
24	F		AGGTTCTTGGGCGGTATTCCG	Ala86 (wt)
25			AGGTTCTTGGACGGTATTCCG	Thr86
26	G		CAACGACATCCACTACAAATT	His105 (wt)
27			CAACGACATCTACTACAAATT	Tyr105
28	H	*mtrD*	TATCCCTCGATGAAGCTGTCC	Ser821/Lys823 (wt)
29			TATCCCTCGATGGAACTGTCC	Ser821/Glu823
30			TATCCCGCTATGGAACTGTCC	Ala821/Glu823 (mosaic)
31			TATCCCGCTATGAAGCTGTCC	Ala821/Lys823
32			TATCCCTCGATGGATCTGTCC	Ser821/Asp823
33			TATCCCGCTATGGATCTGTCC	Ala821/Asp823 (mosaic)
–		Empty gel	-	-
–			-	-
–	M	-	-	Marker elements

Multiplex PCR

Amplification was carried out in two rounds. Reaction mixtures were prepared: mixture (1) for the amplification of the *mtrR* and *mtrD* genes, mixture (2) for the amplification of the *rrnA* operon (23S RNA), mixture (3) – for *rrnB* operon, mixture (4) – for *rrnC* operon, mixture (5) – for *rrnD* operon. The mixtures contained DNA polymerase (SibEnzyme, Russia), dNTPs (Evrogen, Russia), fluorescently labeled Cy5-dUTP (Lumiprobe, Russia), and the corresponding forward and reverse primers (see the Table below). For the 1st round of amplification, *N. gonorrhoeae* genomic DNA was used as a template (1 µl per mixture). PCR was carried out in an S1000 thermal cycler (Bio-Rad, USA) under the following conditions: denaturation for 5 min at 95°C; 29 cycles at 95°C for 30 s, 67°C for 30 s, and 72°C for 30 s; 44 cycles at 95°C for 30 s, 54°C for 30 s, and 72°C for 5 min.

For the second round of amplification, PCR fragments obtained at the first amplification round (1 µl each) were used as a template. Forward and reverse primers for the 2nd round of amplification were added to the mixtures. Reverse primers were added in excess to obtain predominantly single-stranded DNA fragments. The PCR product mixtures after the 2-nd round of amplification containing predominantly single-stranded fluorescently labeled fragments were combined and used for hybridization on microarrays.

First amplification round

	Gene	Direction	Sequence, 5' - 3'	Concentration in the reaction mixture (mM)	Amplicon length (bp)
1st set	*mtrD*	for	CGTATGCAGCCTGCCGA	9.6	228
		rev	TGTAACCGCCGCCCAATT	9.6	
2nd set	*mtrR*	for	CGAACGGGTTGCAAAGCAG	1.0	149
		rev	TGGCGTTTTCGTTTCGGGTC	1.0	
		rev	TGGCGTTTTCGTTCCGTCGC	4.1	
3rd set	*mtrR*	for	AAGAACACCTGATGCTTGCCG	15.0	170
		rev	GTCGTCGCAGATACGTTGGAA	15.0	
	mtrR	for	TCGAAAACTGCATCGCGCAA	15.0	202
		rev	CCAGATTGCCTGATGCTTGC	15.0	
4rd set	*23s rRNA (rrnA)*	for	TTCCCTCTTGACAACGGACG	10.0	3657
		rev	TGCCGATACGGGAGAACCTA	10.0	
	23s rRNA (rrnB)	for	TTCCCTCTTGACAACGGACG	10.0	3660
		rev	TATACGCCGCAATACTGCCC	10.0	
	23s rRNA (rrnC)	for	TTCCCTCTTGACAACGGACG	10.0	3715
		rev	GAAGCCGTATTCGGCAAACC	10.0	
	23s rRNA (rrnD)	for	TTCCCTCTTGACAACGGACG	10.0	3686
		rev	ATGCGGCTGTTCATCCTTGA	10.0	

Second amplification round

	Gene	Direction	Sequence, 5' - 3'	Concentration in the reaction mixture (mM)	Amplicon length (bp)
1st set	*mtrD*	for	CGTATGCAGCCTGCCGA	1.2	228
		rev	TGTAACCGCCGCCCAATT	11.7	
2nd set	*mtrR*	for	CGAACGGGTTGCAAAGCAG	0.3	149
		rev	TGGCGTTTTCGTTTCGGGTC	8.7	
		rev	TGGCGTTTTCGTTCCGTCGC	12.2	
3rd set	*mtrR*	for	AAGAACACCTGATGCTTGCCG	0.7	170
		rev	GTCGTCGCAGATACGTTGGAA	7.0	
		for	TCGAAAACTGCATCGCGCAA	0.7	202
		rev	CCAGATTGCCTGATGCTTGC	7.0	
		rev	CTTTGCAGCCGCTCGAAAAAG	10.5	
4rd set	*23s rRNA (rrnABCD)*	for	CCGAGACTCAGCGAAGTTGA	1.0	710
		rev	GCAACTGGCGTTACAACCG	10.0	

Microarray hybridization

Multiplex PCR product mixture (20 μL) was added to 10 μL of hybridization buffer (0.3 M HEPES (pH 7.5), 3.0 M guanidine thiocyanate, 30 mM EDTA). Microarray hybridization chambers were filled with the mixtures (30 μL), and microarrays were incubated at 37°C for 6-12 hours. After that the hybridization

chambers were removed and the microarray surfaces were washed three times with distilled water and air dried.
