# Supplementary material for: Emergence of Azithromycin-Resistant Neisseria gonorrhoeae Isolates Belonging to the NG-MAST Genogroup 12302 in Russia
Source: Microorganisms. 2023 May 6;11(5):1226. doi: 10.3390/microorganisms11051226 (PMC10222849; doi:10.3390/microorganisms11051226)
Supplement: Supplementary file 1 [file microorganisms-11-01226-s001.zip › Table S1.pdf]

Table S1. Characteristics of *N. gonorrhoeae* isolates collected in the Russian Federation in 2018-2021.

№	Year	Sample code	Ceftriaxone	Azithromycin		23s *rRNA*		*mtrR profile*									*mtrD profile*			NG-MAST	
			MIC_{cro} mg/L	MIC_{azm} mg/L	S/R azith	2058/2059	2611	−35 mosaic	−35 mosaic/delA	−35 delA	−10 insT	Ala39 Thr	Gly45 Asp	Gly45 Ser	Ala86 Thr	His105 Tyr	mosaic	Ser821 Ala	Lys823 Glu	ST	Genogroup
1	2018	10500	0.008	0.12	S	-	-	-	-	-	-	-	+	-	+	-	-	-	-	9570	G10800
2	2018	10501	0.008	0.12	S	-	-	-	-	-	-	+	-	+	+	-	-	-	-	1993	G1993
3	2018	10502	0.004	0.25	S	-	-	-	-	-	-	+	-	-	+	-	-	-	-	17017	G387
4	2018	10503	0.008	0.25	S	-	-	-	-	-	-	-	-	-	+	-	-	-	-	9570	G10800
5	2018	10504	0.03	0.25	S	-	-	-	-	+	-	-	-	-	+	+	-	-	-	10025	G1407
6	2018	10505	0.008	0.25	S	-	-	-	-	-	-	-	-	-	+	-	-	-	-	9570	G10800
7	2018	10506	0.002	0.25	S	-	-	-	-	-	-	+	-	-	+	-	-	-	-	17017	G387
8	2018	10507	0.008	0.25	S	-	-	-	-	+	-	-	+	-	-	+	-	-	-	17018	-
9	2018	10508	0.002	0.03	S	-	-	-	-	-	-	+	-	+	+	-	-	-	-	1993	G1993
10	2018	10509	0.002	0.06	S	-	-	-	-	-	-	-	-	-	-	+	-	-	-	17528	-
11	2018	10510	0.002	0.06	S	-	-	-	-	-	-	-	-	-	-	+	-	-	-	1580	-
12	2018	10511	0.015	0.25	S	-	-	-	-	+	-	-	-	-	+	+	-	-	-	10025	G1407
13	2018	10512	0.002	0.06	S	-	-	-	-	-	-	-	+	-	+	-	-	-	-	17529	-
14	2018	10513	0.008	0.12	S	-	-	-	-	-	-	-	-	-	-	+	-	-	-	16172	-
15	2018	10514	0.004	0.12	S	-	-	-	-	-	-	-	-	-	-	+	-	-	-	16172	-
16	2018	10515	0.002	0.25	S	-	-	-	-	-	-	+	-	+	+	-	-	-	-	5714	G1993
17	2018	10516	0.004	0.12	S	-	-	-	-	-	-	-	+	-	+	-	-	-	-	14603	-
18	2018	10517	0.002	0.06	S	-	-	-	-	-	-	-	-	-	-	+	-	-	-	14825	G387
19	2018	10518	0.002	0.12	S	-	-	-	-	+	-	-	-	-	+	+	-	-	-	17530	-
20	2018	10519	0.002	0.12	S	-	-	-	-	-	-	+	-	+	+	-	-	-	-	17531	-
21	2018	10520	0.002	0.12	S	-	-	-	-	-	-	-	-	-	-	+	-	-	-	16172	-
22	2018	10521	0.004	0.12	S	-	-	-	-	-	-	-	-	-	-	+	-	-	-	17532	G387
23	2018	10522	0.002	0.015	S	-	-	-	-	+	-	-	+	-	+	-	-	-	-	17529	-
24	2018	10523	0.004	0.06	S	-	-	-	-	-	-	-	+	-	+	-	-	-	-	17533	-
25	2018	10524	0.004	0.06	S	-	-	-	-	-	-	-	-	-	+	-	-	-	-	807	G10800
26	2018	10525	0.008	0.12	S	-	-	-	-	-	-	-	-	-	+	+	-	-	-	14942	G14942
27	2018	10526	0.004	0.03	S	-	-	-	-	-	-	-	-	-	+	-	-	-	-	807	G10800
28	2018	10527	0.002	0.06	S	-	-	-	-	-	-	-	-	-	-	+	-	-	-	9486	G387

№	Year	Sample code	Ceftri-axone	Azithro-mycin		23s *rRNA*		*mtrR profile*									*mtrD profile*			NG-MAST	
			MIC_{cro} mg/L	MIC_{azm} mg/L	S/R azith	2058/ 2059	2611	−35 mosaic	−35 mosaic/ delA	−35 delA	−10 insT	Ala39 Thr	Gly45 Asp	Gly45 Ser	Ala86 Thr	His105 Tyr	mosa-ic	Ser821 Ala	Lys823 Glu	ST	Geno-group
29	2018	10528	0.002	0.03	S	-	-	-	-	-	-	-	-	-	-	+	-	-	-	9486	G387
30	2018	10529	0.004	0.03	S	-	-	-	-	-	-	-	-	-	+	-	-	-	-	807	G10800
31	2018	10530	0.002	0.06	S	-	-	-	-	-	-	-	-	-	-	+	-	-	-	14932	G5042
32	2018	10531	0.004	0.03	S	-	-	-	-	-	-	-	-	-	+	-	-	-	-	228	G10800
33	2018	10532	0.015	0.25	S	-	-	-	-	+	-	-	-	-	+	+	-	-	-	21	G5
34	2018	10533	0.002	0.03	S	-	-	-	-	-	-	-	-	-	+	-	-	-	-	807	G10800
35	2018	10534	0.008	0.25	S	-	-	-	-	-	-	-	-	-	+	-	-	-	-	228	G10800
36	2018	10535	0.002	0.03	S	-	-	-	-	+	-	-	-	-	+	+	-	-	-	15619	G10800
37	2018	10536	0.002	0.12	S	-	-	-	-	-	-	-	-	-	+	+	-	-	-	14942	G14942
38	2018	10537	0.015	0.12	S	-	-	-	-	-	-	-	-	-	+	+	-	-	-	14942	G14942
39	2018	10538	0.008	0.12	S	-	-	-	-	-	-	-	-	-	+	+	-	-	-	14942	G14942
40	2018	10539	0.015	0.12	S	-	-	-	-	-	-	-	-	-	+	+	-	-	-	14942	G14942
41	2018	10540	0.002	0.03	S	-	-	-	-	-	-	-	-	-	-	+	-	-	-	5042	G5042
42	2018	10541	0.002	0.12	S	-	-	-	-	-	-	-	-	-	-	+	-	-	-	5042	G5042
43	2018	10542	0.002	0.12	S	-	-	-	-	-	-	-	-	-	-	+	-	-	-	5042	G5042
44	2018	10543	0.002	0.06	S	-	-	-	-	-	-	-	-	-	-	+	-	-	-	5742	-
45	2018	10544	0.004	0.12	S	-	-	-	-	-	-	-	-	-	+	+	-	-	-	14942	G14942
46	2018	10545	0.002	0.12	S	-	-	-	-	-	-	-	-	-	+	+	-	-	-	16410	G14942
47	2018	10546	0.002	0.06	S	-	-	-	-	-	-	-	-	-	-	+	-	-	-	9486	G387
48	2018	10547	0.008	0.12	S	-	-	-	-	-	-	-	-	-	+	+	-	-	-	14942	G14942
49	2018	10548	0.002	0.06	S	-	-	-	-	-	-	-	-	-	-	+	-	-	-	9486	G387
50	2018	10549	0.002	0.25	S	-	-	-	-	-	-	+	-	+	+	-	-	-	-	16411	G1993
51	2018	10550	0.002	0.25	S	-	-	-	-	-	-	+	-	+	+	-	-	-	-	16411	G1993
52	2018	10551	0.004	0.12	S	-	-	-	-	+	-	-	-	-	+	+	-	-	-	13454	-
53	2018	10552	0.002	0.06	S	-	-	-	-	-	-	-	-	-	-	+	-	-	-	9486	G387
54	2018	10553	0.002	0.06	S	-	-	-	-	-	-	-	-	-	-	+	-	-	-	9486	G387
55	2018	10554	0.002	0.06	S	-	-	-	-	-	-	-	+	-	+	-	-	-	-	16412	-
56	2018	10555	0.002	0.06	S	-	-	-	-	-	-	-	+	-	+	-	-	-	-	16412	-

№	Year	Sample code	Ceftri-axone	Azithro-mycin		23s *rRNA*		*mtrR profile*									*mtrD profile*			NG-MAST	
			MIC$_{cro}$ mg/L	MIC$_{azm}$ mg/L	S/R azith	2058/ 2059	2611	−35 mosaic	−35 mosaic/ delA	−35 delA	−10 insT	Ala39 Thr	Gly45 Asp	Gly45 Ser	Ala86 Thr	His105 Tyr	mosa-ic	Ser821 Ala	Lys823 Glu	ST	Geno-group
57	2018	10556	0.12	0.5	S	-	-	-	-	-	-	-	-	-	+	+	-	-	-	5622	G1407
58	2018	10557	0.08	0.12	S	-	-	-	-	-	-	-	-	-	+	+	-	-	-	14942	G14942
59	2018	10558	0.02	0.12	S	-	-	-	-	-	-	-	-	-	+	+	-	-	-	14019	-
60	2018	10559	0.008	0.12	S	-	-	-	-	+	-	-	-	-	-	+	-	-	-	12542	G12542
61	2018	10560	0.002	0.03	S	-	-	-	-	-	-	+	-	-	+	-	-	-	-	17019	-
62	2018	10561	0.002	0.03	S	-	-	-	-	-	-	+	-	-	+	-	-	-	-	14020	G14020
63	2018	10562	0.008	0.12	S	-	-	-	-	-	-	-	-	-	+	-	-	-	-	9576	G10800
64	2018	10563	0.002	0.12	S	-	-	-	-	-	-	-	-	-	-	+	-	-	-	17020	G5042
65	2018	10564	0.015	0.12	S	-	-	-	-	-	-	+	-	-	+	-	-	-	-	14020	G14020
66	2018	10565	0.015	0.25	S	-	-	-	-	+	-	-	-	-	+	+	-	-	-	17534	-
67	2018	10566	0.008	0.25	S	-	-	-	-	-	-	-	+	-	+	-	-	-	-	16175	-
68	2018	10567	0.002	0.06	S	-	-	-	-	-	-	+	-	-	+	-	-	-	-	14020	G14020
69	2018	10568	0.004	0.5	S	-	-	-	+	-	-	-	-	-	+	-	+	+	+	12302	G12302
70	2018	10569	0.015	0.25	S	-	-	-	-	-	-	-	+	-	+	-	-	-	-	16175	-
71	2018	10570	0.002	0.06	S	-	-	-	-	-	-	+	-	-	+	-	-	-	-	14020	G14020
72	2018	10571	0.002	0.06	S	-	-	-	-	-	-	+	-	-	+	-	-	-	-	14020	G14020
73	2018	10572	0.002	0.06	S	-	-	-	-	-	-	+	-	-	+	-	-	-	-	14020	G14020
74	2018	10573	0.008	0.06	S	-	-	-	-	-	-	+	-	-	+	+	-	-	-	18245	G12818
75	2018	10574	0.008	0.12	S	-	-	-	-	-	-	-	-	-	+	-	-	-	-	9576	G10800
76	2018	10575	0.002	0.06	S	-	-	-	-	-	-	-	-	-	-	+	-	-	-	1152	G387
77	2018	10576	0.008	0.12	S	-	-	-	-	-	-	-	-	-	+	-	-	-	-	18246	G10800
78	2018	10577	0.008	0.12	S	-	-	-	-	-	-	-	-	-	+	-	-	-	-	9576	G10800
79	2018	10578	0.008	0.12	S	-	-	-	-	-	-	-	-	-	+	-	-	-	-	18246	G10800
80	2018	10579	0.002	0.06	S	-	-	-	-	-	-	+	-	-	+	-	-	-	-	14020	G14020
81	2018	10580	0.002	0.06	S	-	-	-	-	-	-	-	-	-	-	+	-	-	-	18247	G1993
82	2018	10581	0.004	0.12	S	-	-	-	-	-	-	+	-	-	+	-	-	-	-	1751	-
83	2018	10582	0.06	0.5	S	-	-	-	-	+	-	-	-	-	-	+	-	-	-	9184	G2400
84	2018	10583	0.03	0.12	S	-	-	-	-	+	-	-	-	-	-	+	-	-	-	12542	G12543

№	Year	Sample code	Ceftriaxone	Azithromycin		23s *rRNA*		*mtrR profile*									*mtrD profile*			NG-MAST	
			MIC$_{cro}$ mg/L	MIC$_{azm}$ mg/L	S/R azith	2058/2059	2611	−35 mosaic	−35 mosaic/delA	−35 delA	−10 insT	Ala39 Thr	Gly45 Asp	Gly45 Ser	Ala86 Thr	His105 Tyr	mosaic	Ser821 Ala	Lys823 Glu	ST	Genogroup
85	2018	10584	0.015	0.12	S	-	-	-	-	+	-	-	-	-	-	+	-	-	-	12542	G12544
86	2018	10585	0.008	0.25	S	-	-	-	-	+	-	-	-	-	+	+	-	-	-	12449	G5042
87	2018	10586	0.015	0.5	S	-	-	-	-	+	-	-	-	-	+	+	-	-	-	16185	G6226
88	2018	10587	0.03	0.25	S	-	-	-	-	-	-	-	-	-	-	-	-	-	-	1451	-
89	2018	10588	0.008	0.12	S	-	-	-	-	-	-	-	-	-	+	-	-	-	-	807	G10800
90	2018	10589	0.004	0.06	S	-	-	-	-	-	-	-	+	-	+	-	-	-	-	17024	-
91	2018	10590	0.015	0.12	S	-	-	-	-	-	-	-	-	-	-	-	-	-	-	1451	-
92	2018	10591	0.015	1	S	-	-	-	-	+	-	-	-	-	+	+	-	-	-	13058	G6226
93	2018	10592	0.06	0.5	S	-	-	-	-	+	-	-	-	-	+	+	-	-	-	3149	G1407
94	2018	10593	0.008	0.25	S	-	-	-	-	-	-	-	-	-	-	+	-	-	-	17021	-
95	2018	10594	0.004	0.25	S	-	-	-	-	-	-	-	-	-	-	+	-	-	-	17021	-
96	2018	10595	0.002	0.12	S	-	-	-	-	-	-	+	-	-	+	-	-	-	-	17022	-
97	2018	10596	0.002	0.25	S	-	-	-	-	-	-	+	-	+	+	-	-	-	-	1993	G1993
98	2018	10597	0.002	0.12	S	-	-	-	-	+	-	-	-	-	-	+	-	-	-	17023	-
99	2018	10598	0.002	0.25	S	-	-	-	-	-	-	-	-	-	-	+	-	-	-	17021	-
100	2018	10599	0.002	0.06	S	-	-	-	-	-	-	-	-	-	-	+	-	-	-	5734	G387
101	2018	10600	0.008	0.06	S	-	-	-	-	-	-	-	+	-	+	-	-	-	-	17024	-
102	2018	10601	0.008	0.25	S	-	-	-	-	+	-	-	-	-	+	+	-	-	-	17025	-
103	2018	10602	0.004	0.25	S	-	-	-	-	+	-	-	-	-	+	+	-	-	-	12449	G5042
104	2018	10603	0.002	0.06	S	-	-	-	-	-	-	-	-	-	+	-	-	-	-	228	G10800
105	2018	10604	0.004	0.25	S	-	-	-	-	+	+	-	-	-	+	+	-	-	-	5185	G387
106	2018	10605	0.002	0.06	S	-	-	-	-	-	-	-	-	-	-	+	-	-	-	5734	G387
107	2018	10606	0.008	0.5	S	-	-	-	-	+	-	-	-	-	+	+	-	-	-	12449	G5042
108	2018	10607	0.002	0.06	S	-	-	-	-	+	-	-	-	-	+	+	-	-	-	17535	-
109	2018	10608	0.008	0.25	S	-	-	-	-	+	-	-	-	-	+	+	-	-	-	12446	-
110	2018	10609	0.004	0.06	S	-	-	-	-	+	-	-	-	-	+	+	-	-	-	13055	G6226
111	2018	10610	0.004	0.12	S	-	-	-	-	-	-	-	-	-	+	-	-	-	-	228	G10800
112	2018	10611	0.004	0.12	S	-	-	-	-	-	-	-	-	-	+	-	-	-	-	228	G10800

№	Year	Sample code	Ceftri-axone	Azithro-mycin		23s *rRNA*		*mtrR profile*									*mtrD profile*			NG-MAST	
			MIC_{cro} mg/L	MIC_{azm} mg/L	S/R azith	2058/ 2059	2611	−35 mosaic	−35 mosaic/ delA	−35 delA	−10 insT	Ala39 Thr	Gly45 Asp	Gly45 Ser	Ala86 Thr	His105 Tyr	mosa-ic	Ser821 Ala	Lys823 Glu	ST	Geno-group
113	2018	10612	0.004	0.06	S	-	-	-	-	-	-	-	-	-	+	-	-	-	-	228	G10800
114	2018	10613	0.004	0.06	S	-	-	-	-	-	-	-	-	-	+	-	-	-	-	228	G10800
115	2018	10614	0.004	0.06	S	-	-	-	-	-	-	-	-	-	+	-	-	-	-	228	G10800
116	2018	10615	0.004	0.12	S	-	-	-	-	-	-	-	-	-	+	-	-	-	-	228	G10800
117	2018	10616	0.004	0.06	S	-	-	-	-	-	-	-	-	-	+	-	-	-	-	228	G10800
118	2018	10617	0.004	0.06	S	-	-	-	-	-	-	-	-	-	+	-	-	-	-	228	G10800
119	2018	10618	0.004	0.12	S	-	-	-	-	-	-	-	-	-	+	-	-	-	-	228	G10800
120	2018	10619	0.004	0.12	S	-	-	-	-	-	-	-	-	-	+	-	-	-	-	228	G10800
121	2018	10620	0.002	0.12	S	-	-	-	-	-	-	-	-	-	+	-	-	-	-	228	G10800
122	2018	10621	0.002	0.06	S	-	-	-	-	-	-	-	-	-	-	+	-	-	-	16177	G10800
123	2018	10622	0.004	0.06	S	-	-	-	-	-	-	-	-	-	+	-	-	-	-	228	G10800
124	2018	10623	0.004	0.06	S	-	-	-	-	-	-	-	-	-	+	-	-	-	-	228	G10800
125	2018	10624	0.008	0.06	S	-	-	-	-	-	-	-	-	-	+	-	-	-	-	228	G10800
126	2018	10625	0.004	0.12	S	-	-	-	-	-	-	-	-	-	+	-	-	-	-	228	G10800
127	2018	10626	0.002	0.12	S	-	-	-	-	-	-	-	-	-	+	-	-	-	-	228	G10800
128	2018	10627	0.002	0.06	S	-	-	-	-	-	-	-	-	-	+	-	-	-	-	228	G10800
129	2018	10628	0.002	0.06	S	-	-	-	-	-	-	-	-	-	+	-	-	-	-	228	G10800
130	2018	10629	0.002	0.06	S	-	-	-	-	-	-	-	-	-	+	-	-	-	-	228	G10800
131	2018	10630	0.008	0.25	S	-	-	-	-	-	-	-	-	-	+	+	-	-	-	6226	G6226
132	2018	10631	0.008	0.25	S	-	-	-	-	-	-	-	-	-	+	+	-	-	-	6226	G6226
133	2018	10632	0.004	0.12	S	-	-	-	-	-	-	-	-	-	+	+	-	-	-	6226	G6226
134	2018	10633	0.008	0.12	S	-	-	-	-	-	-	-	-	-	+	+	-	-	-	17026	G6226
135	2018	10634	0.002	0.12	S	-	-	-	-	-	-	-	-	-	-	+	-	-	-	4568	-
136	2018	10635	0.008	0.5	S	-	-	-	-	-	-	-	-	-	+	+	-	-	-	6226	G6226
137	2018	10636	0.002	0.25	S	-	-	-	-	-	-	+	-	+	+	-	-	-	-	1993	G1993
138	2018	10637	0.002	0.06	S	-	-	-	-	-	-	-	-	-	-	+	-	-	-	4568	-
139	2018	10638	0.008	0.25	S	-	-	-	-	-	-	-	-	-	+	-	-	-	-	807	G10800
140	2018	10639	0.002	0.5	S	-	-	-	-	-	-	+	-	+	+	-	-	-	-	1993	G1993

№	Year	Sample code	Ceftri-axone	Azithro-mycin		23s *rRNA*		*mtrR profile*									*mtrD profile*			NG-MAST	
			MIC$_{cro}$ mg/L	MIC$_{azm}$ mg/L	S/R azith	2058/ 2059	2611	−35 mosaic	−35 mosaic/ delA	−35 delA	−10 insT	Ala39 Thr	Gly45 Asp	Gly45 Ser	Ala86 Thr	His105 Tyr	mosa-ic	Ser821 Ala	Lys823 Glu	ST	Geno-group
141	2018	10640	0.008	0.25	S	-	-	-	-	-	-	-	-	-	+	+	-	-	-	17027	G1993
142	2018	10641	0.008	0.25	S	-	-	-	-	-	-	-	-	-	+	+	-	-	-	17027	G1993
143	2018	10642	0.008	0.25	S	-	-	-	-	-	-	-	-	-	+	+	-	-	-	17027	G1993
144	2018	10643	0.002	0.12	S	-	-	-	-	-	-	+	-	+	+	-	-	-	-	14627	G1993
145	2018	10644	0.002	0.25	S	-	-	-	-	-	-	+	-	+	+	-	-	-	-	14627	G1993
146	2018	10645	0.004	0.12	S	-	-	-	-	-	-	-	-	-	+	-	-	-	-	807	G10800
147	2018	10646	0.002	0.25	S	-	-	-	-	-	-	+	-	+	+	-	-	-	-	1993	G1993
148	2018	10647	0.002	0.25	S	-	-	-	-	-	-	+	-	+	+	-	-	-	-	1993	G1993
149	2018	10648	0.015	0.12	S	-	-	-	-	-	-	-	-	-	+	+	-	-	-	6226	G6226
150	2018	10649	0.004	0.06	S	-	-	-	-	-	-	-	-	-	+	-	-	-	-	17536	-
151	2018	10650	0.008	0.12	S	-	-	-	-	-	-	-	-	-	+	-	-	-	-	17536	-
152	2019	10700	0.03	0.5	S	-	-	-	-	+	-	-	-	-	+	+	-	-	-	10025	G1407
153	2019	10701	0.12	0.25	S	-	-	-	-	-	-	-	-	-	+	+	-	-	-	10025	G1407
154	2019	10702	0.004	0.12	S	-	-	-	-	-	-	-	-	-	+	-	-	-	-	9570	G10800
155	2019	10703	0.002	0.12	S	-	-	-	-	-	-	-	-	-	+	-	-	-	-	9570	G10800
156	2019	10704	0.004	0.12	S	-	-	-	-	-	-	-	-	-	+	-	-	-	-	228	G10800
157	2019	10705	0.008	0.25	S	-	-	-	-	+	-	-	+	-	+	-	-	-	-	19649	-
158	2019	10706	0.002	0.03	S	-	-	-	-	-	-	-	-	-	-	+	-	-	-	1580	-
159	2019	10707	0.002	0.03	S	-	-	-	-	-	-	-	-	-	-	+	-	-	-	1580	-
160	2019	10708	0.002	0.25	S	-	-	-	-	-	-	+	-	+	+	-	-	-	-	5714	G1993
161	2019	10709	0.004	0.06	S	-	-	-	-	-	-	-	+	-	+	-	-	-	-	19587	-
162	2019	10710	0.002	0.06	S	-	-	-	-	-	-	-	-	-	-	+	-	-	-	9486	G387
163	2019	10711	0.002	0.03	S	-	-	-	-	-	-	-	-	-	+	-	-	-	-	6967	G10800
164	2019	10712	0.002	0.06	S	-	-	-	-	-	-	-	-	-	-	+	-	-	-	9486	G387
165	2019	10713	0.002	0.06	S	-	-	-	-	-	-	-	-	-	-	+	-	-	-	9486	G387
166	2019	10714	0.015	0.06	S	-	-	-	-	-	-	-	-	-	+	-	-	-	-	18952	-
167	2019	10715	0.002	0.25	S	-	-	-	-	-	-	+	-	-	+	-	-	-	-	18951	-
168	2019	10716	0.002	0.25	S	-	-	-	-	-	-	+	-	-	+	-	-	-	-	18951	-

№	Year	Sample code	Ceftriaxone	Azithromycin		23s *rRNA*		*mtrR profile*									*mtrD profile*			NG-MAST	
			MIC_{cro} mg/L	MIC_{azm} mg/L	S/R azith	2058/2059	2611	−35 mosaic	−35 mosaic/delA	−35 delA	−10 insT	Ala39Thr	Gly45Asp	Gly45Ser	Ala86Thr	His105Tyr	mosaic	Ser821Ala	Lys823Glu	ST	Genogroup
169	2019	10717	0.002	0.12	S	-	-	-	-	-	-	-	-	-	-	+	-	-	-	9486	G387
170	2019	10718	0.002	0.12	S	-	-	-	-	-	-	-	-	-	-	+	-	-	-	9486	G387
171	2019	10719	0.002	0.06	S	-	-	-	-	-	-	-	-	-	-	+	-	-	-	14940	G5042
172	2019	10720	0.002	0.12	S	-	-	-	-	-	-	-	-	-	+	-	-	-	-	1544	G10800
173	2019	10721	0.004	0.12	S	-	-	-	-	-	-	+	-	-	+	-	-	-	-	16169	-
174	2019	10722	0.002	0.06	S	-	-	-	-	-	-	-	-	-	-	+	-	-	-	9486	G387
175	2019	10723	0.004	0.06	S	-	-	-	-	-	-	-	-	-	+	-	-	-	-	807	G10800
176	2019	10724	0.004	0.25	S	-	-	-	-	-	-	-	-	-	+	-	-	-	-	1544	G10800
177	2019	10725	0.004	0.06	S	-	-	-	-	-	-	-	-	-	+	-	-	-	-	807	G10800
178	2019	10726	0.004	0.06	S	-	-	-	-	-	-	-	-	-	-	+	-	-	-	5042	G5042
179	2019	10727	0.004	0.03	S	-	-	-	-	-	-	-	-	-	+	-	-	-	-	228	G10800
180	2019	10728	0.004	0.12	S	-	-	-	-	+	-	-	-	-	+	+	-	-	-	13454	-
181	2019	10729	0.002	0.015	S	-	-	-	-	-	-	-	+	-	+	-	-	-	-	16412	-
182	2019	10730	0.002	0.06	S	-	-	-	-	-	-	-	-	-	-	+	-	-	-	14940	G5042
183	2019	10731	0.002	0.25	S	-	-	-	-	-	-	+	-	+	+	-	-	-	-	1993	G1993
184	2019	10732	0.002	0.06	S	-	-	-	-	-	-	-	-	-	-	+	-	-	-	9486	G387
185	2019	10733	0.002	0.12	S	-	-	-	-	-	-	-	-	-	+	-	-	-	-	1544	G10800
186	2019	10734	0.002	0.06	S	-	-	-	-	-	-	+	-	+	+	-	-	-	-	19573	G10800
187	2019	10735	0.002	0.06	S	-	-	-	-	-	-	-	-	-	+	+	-	-	-	14940	G5042
188	2019	10736	0.002	0.06	S	-	-	-	-	-	-	+	-	+	+	-	-	-	-	1993	G1993
189	2019	10737	0.008	0.03	S	-	-	-	-	-	-	-	+	-	+	-	-	-	-	16412	-
190	2019	10738	0.002	0.06	S	-	-	-	-	-	-	+	-	+	+	-	-	-	-	1993	G1993
191	2019	10739	0.004	0.06	S	-	-	-	-	-	-	+	-	-	+	-	-	-	-	14020	G14020
192	2019	10740	0.002	0.03	S	-	-	-	-	-	-	-	-	-	-	+	-	-	-	9574	G5042
193	2019	10741	0.015	0.06	S	-	-	-	-	-	-	-	-	-	+	-	-	-	-	18894	G10800
194	2019	10742	0.015	0.06	S	-	-	-	-	-	-	-	-	-	+	-	-	-	-	18894	G10800
195	2019	10743	0.002	0.12	S	-	-	-	-	-	-	+	-	+	+	-	-	-	-	5714	G1993
196	2019	10744	0.002	0.06	S	-	-	-	-	-	-	+	-	+	+	-	-	-	-	5714	G1993

№	Year	Sample code	Ceftri-axone	Azithro-mycin		23s *rRNA*		*mtrR profile*									*mtrD profile*			NG-MAST	
			MICcro mg/L	MICazm mg/L	S/R azith	2058/ 2059	2611	−35 mosaic	−35 mosaic/ delA	−35 delA	−10 insT	Ala39 Thr	Gly45 Asp	Gly45 Ser	Ala86 Thr	His105 Tyr	mosa-ic	Ser821 Ala	Lys823 Glu	ST	Geno-group
197	2019	10745	0.008	0.06	S	-	-	-	-	+	-	+	-	-	+	-	-	-	-	19575	-
198	2019	10746	0.004	0.06	S	-	-	-	-	-	-	+	-	-	+	-	-	-	-	14020	G14020
199	2019	10747	0.015	0.12	S	-	-	-	-	+	-	-	-	-	+	+	-	-	-	19574	G5441
200	2019	10748	0.002	0.015	S	-	-	-	-	-	-	+	-	+	+	-	-	-	-	5714	G1993
201	2019	10749	0.008	0.06	S	-	-	-	-	-	-	-	-	-	-	+	-	-	-	19648	-
202	2019	10750	0.008	0.03	S	-	-	-	-	-	-	-	-	-	+	-	-	-	-	18894	G10800
203	2019	10751	0.002	0.06	S	-	-	-	-	-	-	+	-	+	+	-	-	-	-	16174	G1993
204	2019	10752	0.002	0.06	S	-	-	-	-	-	-	-	-	-	-	+	-	-	-	19576	G19572
205	2019	10753	0.004	0.06	S	-	-	-	-	-	-	-	-	-	+	-	-	-	-	18894	G10800
206	2019	10754	0.004	0.06	S	-	-	-	-	+	-	-	-	-	+	+	-	-	-	17534	-
207	2019	10756	0.008	0.25	S	-	-	-	-	-	-	+	-	-	+	-	-	-	-	18948	-
208	2019	10757	0.03	0.25	S	-	-	-	-	-	-	+	-	-	+	-	-	-	-	18948	-
209	2019	10758	0.015	0.5	S	-	-	-	-	-	-	+	-	-	+	-	-	-	-	18948	-
210	2019	10759	0.015	0.12	S	-	-	-	-	-	-	+	-	-	+	-	-	-	-	18948	-
211	2019	10760	0.015	0.12	S	-	-	-	-	-	-	+	-	-	+	-	-	-	-	18948	-
212	2019	10761	0.008	0.12	S	-	-	-	-	-	-	+	-	-	+	-	-	-	-	18948	-
213	2019	10762	0.002	0.06	S	-	-	-	-	-	-	-	-	-	-	+	-	-	-	19578	-
214	2019	10763	0.002	0.06	S	-	-	-	-	-	-	-	-	-	-	+	-	-	-	19578	-
215	2019	10764	0.002	0.06	S	-	-	-	-	-	-	-	-	-	-	+	-	-	-	19578	-
216	2019	10765	0.002	0.03	S	-	-	-	-	-	-	-	-	-	-	+	-	-	-	19578	-
217	2019	10766	0.002	0.06	S	-	-	-	-	+	-	-	-	-	+	+	-	-	-	19578	-
218	2019	10767	0.002	0.06	S	-	-	-	-	-	-	-	-	-	-	+	-	-	-	19578	-
219	2019	10768	0.03	0.5	S	-	-	-	+	-	-	-	-	-	+	-	+	+	+	15906	-
220	2019	10769	0.008	0.25	S	-	-	-	-	-	-	+	-	-	+	-	-	-	-	16169	-
221	2019	10770	0.008	0.06	S	-	-	-	+	-	-	-	-	-	+	-	+	+	+	6765	G12302
222	2019	10771	0.03	0.12	S	-	-	-	-	-	-	-	-	-	+	-	-	-	-	807	G10800
223	2019	10772	0.03	0.12	S	-	-	-	-	-	-	-	-	-	+	-	-	-	-	807	G10800
224	2019	10773	0.004	0.06	S	-	-	-	-	+	-	-	-	-	+	+	-	-	-	13055	G6226

№	Year	Sample code	Ceftri-axone	Azithro-mycin		23s *rRNA*		*mtrR profile*									*mtrD profile*			NG-MAST	
			MIC_{cro} mg/L	MIC_{azm} mg/L	S/R azith	2058/ 2059	2611	−35 mosaic	−35 mosaic/ delA	−35 delA	−10 insT	Ala39 Thr	Gly45 Asp	Gly45 Ser	Ala86 Thr	His105 Tyr	mosa-ic	Ser821 Ala	Lys823 Glu	ST	Geno-group
225	2019	10774	0.002	0.06	S	-	-	-	-	-	-	-	-	-	-	-	-	-	-	18895	-
226	2019	10775	0.015	0.12	S	-	-	-	-	-	-	-	-	-	+	-	-	-	-	807	G10800
227	2019	10776	0.015	0.25	S	-	-	-	-	+	-	-	-	-	+	+	-	-	-	13055	G6226
228	2019	10777	0.002	0.06	S	-	-	-	-	-	-	-	+	-	+	-	-	-	-	17024	-
229	2019	10778	0.004	0.06	S	-	-	-	-	-	-	-	-	-	-	+	-	-	-	18950	-
230	2019	10779	0.015	0.06	S	-	-	-	-	-	-	-	-	-	+	-	-	-	-	807	G10800
231	2019	10780	0.002	0.06	S	-	-	-	-	-	-	-	-	-	-	+	-	-	-	17535	-
232	2019	10781	0.008	0.5	S	-	-	-	-	+	-	-	-	-	+	+	-	-	-	13058	G6226
233	2019	10782	0.004	0.03	S	-	-	-	-	-	-	-	+	-	+	-	-	-	-	17024	-
234	2019	10783	0.015	0.5	S	-	-	-	-	+	-	-	-	-	+	+	-	-	-	1318	-
235	2019	10784	0.015	0.25	S	-	-	-	-	-	-	-	+	-	+	-	-	-	-	17024	-
236	2019	10785	0.002	0.03	S	-	-	-	-	-	-	-	-	-	-	+	-	-	-	19577	-
237	2019	10786	0.015	0.25	S	-	-	-	-	-	-	+	-	-	+	-	-	-	-	16169	-
238	2019	10787	0.004	0.03	S	-	-	-	-	-	-	-	-	-	+	-	-	-	-	228	G10800
239	2019	10788	0.002	0.002	S	-	-	-	-	-	-	-	-	-	-	-	-	-	-	18895	-
240	2019	10789	0.004	0.06	S	-	-	-	-	+	-	-	-	-	+	+	-	-	-	13055	G6226
241	2019	10790	0.004	0.03	S	-	-	-	-	-	-	-	-	-	+	-	-	-	-	807	G10800
242	2019	10791	0.008	0.25	S	-	-	-	-	-	-	-	-	-	+	-	-	-	-	5941	G10800
243	2019	10792	0.004	0.25	S	-	-	-	-	-	-	-	-	-	+	-	-	-	-	807	G10800
244	2019	10793	0.008	0.12	S	-	-	-	-	-	-	-	-	-	+	-	-	-	-	807	G10800
245	2019	10794	0.008	0.12	S	-	-	-	-	-	-	-	-	-	+	-	-	-	-	9570	G10800
246	2019	10795	0.004	0.06	S	-	-	-	-	-	-	-	-	-	+	-	-	-	-	5941	G10800
247	2019	10796	0.002	0.12	S	-	-	-	-	-	-	+	-	+	+	-	-	-	-	1993	G1993
248	2019	10797	0.004	0.06	S	-	-	-	-	-	-	-	-	-	+	-	-	-	-	5941	G10800
249	2019	10798	0.002	0.25	S	-	-	-	-	-	-	+	-	+	+	-	-	-	-	14627	G1993
250	2019	10799	0.008	0.06	S	-	-	-	-	-	-	-	-	-	+	-	-	-	-	18953	G4186
251	2019	10800	0.008	0.25	S	-	-	-	-	-	-	-	-	-	+	-	-	-	-	807	G10800
252	2019	10801	0.002	0.12	S	-	-	-	-	-	-	-	-	+	+	-	-	-	-	1993	G1993

№	Year	Sample code	Ceftriaxone	Azithromycin		23s *rRNA*		*mtrR profile*									*mtrD profile*			NG-MAST	
			MIC_{cro} mg/L	MIC_{azm} mg/L	S/R azith	2058/2059	2611	−35 mosaic	−35 mosaic/delA	−35 delA	−10 insT	Ala39 Thr	Gly45 Asp	Gly45 Ser	Ala86 Thr	His105 Tyr	mosaic	Ser821 Ala	Lys823 Glu	ST	Genogroup
253	2019	10802	0.002	0.03	S	-	-	-	-	-	-	-	-	-	+	-	-	-	-	5926	-
254	2019	10803	0.008	0.06	S	-	-	-	-	-	-	-	-	-	+	+	-	-	-	6226	G6226
255	2019	10804	0.004	0.03	S	-	-	-	-	-	-	-	-	-	+	+	-	-	-	6226	G6226
256	2019	10805	0.002	0.12	S	-	-	-	-	-	-	-	-	-	-	+	-	-	-	5718	-
257	2019	10806	0.008	0.12	S	-	-	-	-	-	-	-	-	-	+	+	-	-	-	17027	G1993
258	2019	10807	0.008	0.12	S	-	-	-	-	-	-	-	-	-	+	-	-	-	-	19572	G19572
259	2019	10808	0.008	0.12	S	-	-	-	-	-	-	-	-	-	+	+	-	-	-	6226	G6226
260	2019	10809	0.008	0.06	S	-	-	-	-	-	-	-	+	-	+	+	-	-	-	6238	G6226
261	2019	10810	0.008	0.06	S	-	-	-	-	-	-	-	-	-	-	-	-	-	-	19588	G799
262	2019	10811	0.008	0.12	S	-	-	-	-	-	-	-	+	-	+	-	-	-	-	18953	G4186
263	2019	10812	0.008	0.06	S	-	-	-	-	-	-	-	+	-	-	-	-	-	-	19588	G799
264	2019	10813	0.002	0.5	S	-	-	-	-	-	-	-	-	+	+	-	-	-	-	1993	G1993
265	2019	10814	0.004	0.06	S	-	-	-	-	-	-	-	-	-	+	-	-	-	-	228	G10800
266	2019	10815	0.002	0.06	S	-	-	-	-	-	-	-	-	-	-	+	-	-	-	19589	-
267	2019	10816	0.004	0.12	S	-	-	-	-	-	-	-	-	-	+	-	-	-	-	19572	G19572
268	2019	10817	0.002	0.25	S	-	-	-	-	-	-	-	-	+	+	-	-	-	-	1993	G1993
269	2019	10818	0.008	0.12	S	-	-	-	-	-	-	-	-	-	+	-	-	-	-	19572	G19572
270	2019	10819	0.002	0.12	S	-	-	-	-	-	-	+	+	-	+	-	-	-	-	1993	G1993
271	2019	10820	0.008	0.12	S	-	-	-	-	-	-	+	-	-	+	-	-	-	-	19650	-
272	2019	10821	0.002	0.12	S	-	-	-	-	-	-	-	-	+	+	-	-	-	-	1993	G1993
273	2019	10822	0.008	0.06	S	-	-	-	-	-	-	-	-	-	+	-	-	-	-	19650	-
274	2020	10900	0.015	4	R	-	-	-	+	-	-	-	-	-	+	-	+	+	+	20602	G12302
275	2020	10901	0.008	0.06	S	-	-	-	-	-	-	-	-	-	+	-	-	-	-	3369	G10800
276	2020	10902	0.03	0.5	S	-	-	-	-	+	-	-	-	-	+	+	-	-	-	2212	G1407
277	2020	10903	0.004	4	R	-	-	-	+	-	-	-	-	-	+	-	+	+	+	17380	G12302
278	2020	10904	0.03	0.5	S	-	-	-	-	+	-	-	-	-	+	+	-	-	-	2212	G1407
279	2020	10909	0.015	4	R	-	-	-	+	-	-	-	-	-	+	-	+	+	+	17380	G12302
280	2020	10910	0.008	0.06	S	-	-	-	-	-	-	-	-	-	+	-	-	-	-	4570	G10800

№	Year	Sample code	Ceftriaxone	Azithromycin		23s *rRNA*		*mtrR profile*									*mtrD profile*			NG-MAST	
			MIC_{cro} mg/L	MIC_{azm} mg/L	S/R azith	2058/ 2059	2611	−35 mosaic	−35 mosaic/ delA	−35 delA	−10 insT	Ala39 Thr	Gly45 Asp	Gly45 Ser	Ala86 Thr	His105 Tyr	mosaic	Ser821 Ala	Lys823 Glu	ST	Genogroup
281	2020	10911	0.004	0.12	S	-	-	-	-	-	-	-	-	-	+	-	-	-	-	5941	G10800
282	2020	10913	0.008	0.25	S	-	-	-	-	-	-	+	-	-	+	-	-	-	-	17375	-
283	2020	10914	0.008	4	R	-	+	-	+	-	-	-	-	-	+	-	+	+	+	17380	G12302
284	2020	10915	0.008	4	R	-	-	-	+	-	-	-	-	-	+	-	+	+	+	20602	G12302
285	2020	10918	0.015	4	R	-	-	-	+	-	-	-	-	-	+	-	+	+	+	20602	G12302
286	2020	10920	0.004	0.12	S	-	-	-	-	-	-	-	-	-	+	-	-	-	-	807	G10800
287	2020	10921	0.004	0.12	S	-	-	-	-	-	-	-	-	-	+	-	-	-	-	4570	G10800
288	2020	10922	0.002	0.06	S	-	-	-	-	-	-	-	-	-	+	-	-	-	-	5941	G10800
289	2020	10923	0.004	0.12	S	-	-	-	-	-	-	-	-	-	+	-	-	-	-	807	G10800
290	2020	10924	0.008	4	R	-	-	-	+	-	-	-	-	-	+	-	+	+	+	12302	G12302
291	2020	10925	0.008	4	R	-	-	-	+	-	-	-	-	-	+	-	+	+	+	12302	G12302
292	2020	10926	0.008	2	R	-	-	-	+	-	-	-	-	-	+	-	+	+	+	12302	G12302
293	2020	10927	0.004	2	R	-	-	-	+	-	-	-	-	-	+	-	+	+	+	12302	G12302
294	2020	10928	0.002	0.06	S	-	-	-	-	-	-	-	-	-	+	-	-	-	-	807	G10800
295	2020	10929	0.002	0.06	S	-	-	-	-	-	-	-	-	-	+	-	-	-	-	807	G10800
296	2020	10930	0.008	0.25	S	-	-	-	-	-	-	-	-	-	+	-	-	-	-	3321	G10800
297	2020	10932	0.002	0.06	S	-	-	-	-	-	-	-	-	-	+	-	-	-	-	807	G10800
298	2020	10933	0.004	0.06	S	-	-	-	-	-	-	-	-	-	+	-	-	-	-	807	G10800
299	2020	10934	0.002	0.25	S	-	-	-	-	-	-	+	-	+	+	-	-	-	-	1993	G1993
300	2020	10935	0.004	0.06	S	-	-	-	-	-	-	+	-	+	+	-	-	-	-	228	G10800
301	2020	10937	0.008	0.06	S	-	-	-	-	-	-	-	-	-	+	-	-	-	-	228	G10800
302	2020	10938	0.002	0.06	S	-	-	-	-	-	-	-	-	-	+	-	-	-	-	807	G10800
303	2020	10939	0.002	0.06	S	-	-	-	-	-	-	-	-	-	+	-	-	-	-	228	G10800
304	2020	10940	0.008	4	R	-	-	-	-	-	-	+	-	-	+	-	+	+	+	12302	G12302
305	2020	10941	0.002	0.06	S	-	-	-	-	-	-	+	-	+	+	-	-	-	-	1993	G1993
306	2020	10942	0.004	0.12	S	-	-	-	-	-	-	-	-	-	+	-	-	-	-	807	G10800
307	2020	10943	0.008	0.12	S	-	-	-	-	-	-	-	-	-	+	-	-	-	-	19572	G19572
308	2020	10944	0.004	0.12	S	-	-	-	-	-	-	-	-	-	+	-	-	-	-	807	G10800

№	Year	Sample code	Ceftri-axone	Azithro-mycin		23s *rRNA*		*mtrR profile*									*mtrD profile*			NG-MAST	
			MIC_{cro} mg/L	MIC_{azm} mg/L	S/R azith	2058/2059	2611	−35 mosaic	−35 mosaic/delA	−35 delA	−10 insT	Ala39 Thr	Gly45 Asp	Gly45 Ser	Ala86 Thr	His105 Tyr	mosa-ic	Ser821 Ala	Lys823 Glu	ST	Geno-group
309	2020	10945	0.002	0.12	S	-	-	-	-	-	-	+	-	+	+	-	-	-	-	1993	G1993
310	2020	10946	0.002	0.06	S	-	-	-	-	-	-	+	-	+	+	-	-	-	-	1993	G1993
311	2020	10947	0.004	0.12	S	-	-	-	-	-	-	-	-	-	+	+	-	-	-	6226	G6226
312	2020	10948	0.002	0.03	S	-	-	-	-	-	-	-	-	-	+	-	-	-	-	228	G10800
313	2020	10949	0.015	0.25	S	-	-	-	-	+	-	-	+	-	+	-	-	-	-	14700	G14700
314	2020	10950	0.015	0.12	S	-	-	-	-	-	-	+	-	-	+	-	-	-	-	15589	G15589
315	2020	10951	0.008	8	R	-	-	-	-	-	-	-	-	-	+	-	+	+	+	17380	G12302
316	2020	10952	0.002	0.12	S	-	-	-	-	-	-	-	+	-	+	-	-	-	-	5792	-
317	2020	10953	0.004	8	R	-	-	-	-	-	-	-	+	-	+	-	+	+	+	17380	G12302
318	2020	10954	0.004	8	R	-	-	-	+	-	-	-	-	-	+	-	+	+	+	17380	G12302
319	2020	10955	0.004	0.06	S	-	-	-	+	-	-	-	-	-	+	-	+	+	+	12302	G12302
320	2020	10956	0.015	0.06	S	-	-	-	-	-	-	-	-	-	+	-	-	-	-	807	G10800
321	2020	10957	0.002	0.03	S	-	-	-	-	-	-	-	+	-	+	-	-	-	-	20617	-
322	2020	10958	0.002	0.06	S	-	-	-	-	-	-	+	-	+	+	-	-	-	-	20614	G9918
323	2020	10959	0.002	0.06	S	-	-	-	-	-	-	+	-	+	+	-	-	-	-	1993	G1993
324	2020	10960	0.002	0.06	S	-	-	-	-	-	-	-	-	-	+	-	-	-	-	228	G10800
325	2020	10961	0.008	0.5	S	-	-	-	-	+	-	-	+	-	+	-	-	-	-	14700	G14700
326	2020	10962	0.004	0.25	S	-	-	-	-	-	-	-	-	-	+	-	-	-	-	3321	G10800
327	2020	10963	0.015	0.12	S	-	-	-	-	-	-	+	-	-	+	-	-	-	-	15589	G15589
328	2020	10966	0.008	0.25	S	-	-	-	-	-	-	+	-	-	+	-	-	-	-	18898	-
329	2020	10967	0.004	0.12	S	-	-	-	-	-	-	+	-	-	+	-	-	-	-	18898	-
330	2020	10968	0.004	0.5	S	-	-	-	-	-	-	+	-	-	+	-	-	-	-	18898	-
331	2020	10969	0.004	0.12	S	-	-	-	-	-	-	-	-	-	+	-	-	-	-	807	G10800
332	2020	10970	0.002	0.12	S	-	-	-	-	-	-	-	-	-	+	-	-	-	-	17020	G5042
333	2020	10981	-	0.12	S	-	-	-	-	-	-	-	-	-	+	-	-	-	-	16174	G1993
334	2020	10982	0.008	0.12	S	-	-	-	-	+	-	-	+	-	+	-	-	-	-	20611	-
335	2020	10983	0.008	0.12	S	-	-	-	-	-	-	-	-	-	+	-	-	-	-	18894	G10800
336	2020	10984	0.004	0.12	S	-	-	-	-	+	-	+	-	-	+	-	-	-	-	18131	G3785

№	Year	Sample code	Ceftriaxone	Azithromycin		23s *rRNA*		*mtrR profile*									*mtrD profile*			NG-MAST	
			MIC$_{cro}$ mg/L	MIC$_{azm}$ mg/L	S/R azith	2058/2059	2611	−35 mosaic	−35 mosaic/delA	−35 delA	−10 insT	Ala39 Thr	Gly45 Asp	Gly45 Ser	Ala86 Thr	His105 Tyr	mosaic	Ser821 Ala	Lys823 Glu	ST	Genogroup
337	2020	10988	0.015	0.12	S	-	-	-	-	-	-	+	-	-	+	-	-	-	-	20610	-
338	2020	11007	0.002	0.12	S	-	-	-	-	-	-	-	-	-	+	-	-	-	-	20615	G12302
339	2020	11008	0.004	0.03	S	-	-	-	+	-	-	-	-	-	+	-	+	+	+	14502	G12302
340	2020	11009	0.015	0.12	S	-	-	-	-	+	-	-	-	-	+	+	-	-	-	20616	-
341	2020	11010	0.015	0.12	S	-	-	-	-	+	-	-	-	-	+	+	-	-	-	20616	-
342	2020	11012	0.002	0.03	S	-	-	-	-	-	-	-	-	-	-	+	-	-	-	20609	-
343	2020	11014	0.002	0.06	S	-	-	-	-	-	-	-	-	-	-	+	-	-	-	5742	-
344	2020	11015	0.002	0.06	S	-	-	-	-	-	-	-	-	-	-	+	-	-	-	5742	-
345	2020	11016	0.002	0.03	S	-	-	-	-	-	-	-	-	-	-	+	-	-	-	5742	-
346	2020	11018	0.004	0.03	S	-	-	-	+	-	-	-	-	-	+	-	+	+	+	14502	G12302
347	2020	11023	0.004	0.06	S	-	-	-	-	-	-	-	-	-	+	-	-	-	-	17536	-
348	2020	11024	0.03	4	R	-	-	-	-	-	-	-	-	-	+	-	+	+	+	16953	G12302
349	2020	11025	0.03	4	R	-	-	-	-	-	-	-	-	-	+	-	-	-	-	19572	G19572
350	2020	11026	0.002	0.03	S	-	-	-	-	-	-	-	-	-	-	+	-	-	-	5742	-
351	2020	11027	0.002	0.12	S	-	-	-	-	-	-	-	-	-	-	+	-	-	-	5718	-
352	2020	11028	0.008	0.12	S	-	-	-	-	-	-	-	-	-	+	-	-	-	-	20619	-
353	2020	11032	0.06	0.25	S	-	-	-	-	+	-	-	-	-	+	+	-	-	-	2212	G1407
354	2020	11035	0.015	0.25	S	-	-	-	-	-	-	-	-	-	+	-	-	-	-	228	G10800
355	2020	11037	0.002	0.12	S	-	-	-	-	-	-	+	-	+	+	-	-	-	-	20609	-
356	2020	11038	0.008	0.12	S	-	-	-	-	-	-	-	-	-	+	-	-	-	-	807	G10800
357	2020	11039	0.008	4	R	-	+	-	-	-	-	+	-	-	+	-	-	-	-	16169	-
358	2020	11040	0.002	0.06	S	-	-	-	-	-	-	+	-	-	+	-	-	-	-	807	G10800
359	2020	11041	0.002	0.12	S	-	-	-	-	-	-	-	-	-	-	-	-	-	-	807	G10800
360	2020	11042	0.002	0.06	S	-	-	-	-	-	-	-	-	-	-	+	-	-	-	20613	-
361	2020	11043	0.002	0.03	S	-	-	-	-	-	-	+	-	+	+	-	-	-	-	20614	G9918
362	2020	11044	0.002	0.02	S	-	-	-	-	-	-	-	+	-	+	-	-	-	-	20617	-
363	2020	11045	0.008	0.25	S	-	-	-	-	+	-	-	+	-	+	-	-	-	-	20618	-
364	2020	11046	0.004	0.06	S	-	-	-	-	-	-	-	-	-	+	-	-	-	-	17536	-

№	Year	Sample code	Ceftri-axone	Azithro-mycin		23s *rRNA*		*mtrR profile*									*mtrD profile*			NG-MAST	
			MIC cro mg/L	MIC azm mg/L	S/R azith	2058/ 2059	2611	−35 mosaic	−35 mosaic/ delA	−35 delA	−10 insT	Ala39 Thr	Gly45 Asp	Gly45 Ser	Ala86 Thr	His105 Tyr	mosa-ic	Ser821 Ala	Lys823 Glu	ST	Geno-group
365	2020	11047	0.002	0.25	S	-	-	-	-	-	-	-	-	-	-	+	-	-	-	1993	G1993
366	2020	11048	0.008	0.12	S	-	-	-	-	-	-	-	-	-	+	-	-	-	-	17536	-
367	2020	11049	0.004	0.12	S	-	-	-	-	-	-	-	-	-	+	-	-	-	-	807	G10800
368	2020	11050	0.008	0.12	S	-	-	-	-	-	-	-	-	-	+	-	-	-	-	19572	G19572
369	2020	11051	0.008	0.12	S	-	-	-	-	-	-	-	-	-	+	-	-	-	-	228	G10800
370	2020	11052	0.004	0.12	S	-	-	-	-	-	-	-	-	-	+	-	-	-	-	228	G10800
371	2020	11053	0.008	0.12	S	-	-	-	-	-	-	-	-	-	+	-	-	-	-	228	G10800
372	2020	11054	0.002	0.12	S	-	-	-	-	-	-	-	-	-	-	+	-	-	-	5718	-
373	2020	11056	0.004	0.12	S	-	-	-	-	-	-	-	-	-	+	-	-	-	-	807	G10800
374	2020	11057	0.004	0.12	S	-	-	-	-	-	-	-	-	-	+	-	-	-	-	17536	-
375	2021	11100	0.002	0.12	S	-	-	-	-	-	-	+	-	-	+	-	+	+	+	19838	-
376	2021	11101	0.002	0.06	S	-	-	-	-	+	-	-	-	-	+	+	-	-	-	20283	G1407
377	2021	11102	0.015	0.03	S	-	-	-	-	-	-	+	-	-	+	-	-	-	-	20282	-
378	2021	11103	0.002	0.03	S	-	-	-	-	-	-	+	-	-	+	-	+	+	+	20281	-
379	2021	11104	0.002	0.12	S	-	-	-	-	-	-	+	-	+	+	-	-	-	-	20273	G1993
380	2021	11105	0.002	0.12	S	-	-	-	-	-	-	+	-	-	+	-	+	+	+	19838	-
381	2021	11106	0.002	0.25	S	-	-	-	+	-	-	-	-	-	+	-	+	+	+	20294	G12302
382	2021	11107	0.002	0.25	S	-	-	-	-	-	-	+	-	-	+	-	+	+	+	19838	-
383	2021	11108	0.002	0.12	S	-	-	-	-	-	-	+	-	-	+	-	-	-	-	20287	-
384	2021	11109	0.004	0.06	S	-	-	-	-	-	-	+	-	-	+	-	-	-	-	20286	-
385	2021	11110	0.002	0.12	S	-	-	-	-	-	-	-	-	-	-	+	-	-	-	9575	-
386	2021	11111	0.004	0.25	S	-	-	-	-	-	-	+	-	-	+	-	-	-	-	19778	-
387	2021	11112	0.015	0.12	S	-	-	-	-	-	-	+	-	-	+	-	-	-	-	18898	-
388	2021	11113	0.015	0.06	S	-	-	-	-	-	-	+	-	-	+	-	-	-	-	18898	-
389	2021	11114	0.008	0.06	S	-	-	-	-	+	-	-	-	-	+	+	-	-	-	20285	G1407
390	2021	11115	0.004	0.5	S	-	-	-	+	-	-	-	-	-	+	-	+	+	+	20284	-
391	2021	11116	0.015	0.12	S	-	-	-	-	-	-	-	-	-	+	-	+	+	+	20274	G12302
392	2021	11117	0.004	0.5	S	-	-	-	-	-	-	+	-	-	+	-	-	-	-	20289	-

№	Year	Sample code	Ceftri-axone	Azithro-mycin		23s *rRNA*		*mtrR profile*									*mtrD profile*			NG-MAST	
			MIC$_{cro}$ mg/L	MIC$_{azm}$ mg/L	S/R azith	2058/2059	2611	−35 mosaic	−35 mosaic/delA	−35 delA	−10 insT	Ala39 Thr	Gly45 Asp	Gly45 Ser	Ala86 Thr	His105 Tyr	mosa-ic	Ser821 Ala	Lys823 Glu	ST	Geno-group
393	2021	11118	0.008	0.03	S	-	-	-	-	-	-	+	-	-	+	-	-	-	-	13994	G5441
394	2021	11119	0.008	0.12	S	-	-	-	-	-	-	+	-	-	+	-	-	-	-	20269	-
395	2021	11120	0.008	0.25	S	-	-	-	+	-	-	-	-	-	+	-	+	+	+	20288	-
396	2021	11121	0.008	0.01	S	-	-	-	-	-	-	+	-	-	+	-	-	-	-	20269	-
397	2021	11122	0.002	0.02	S	-	-	-	-	-	-	-	-	-	+	-	-	-	-	1152	G387
398	2021	11123	0.002	0.12	S	-	-	-	-	-	-	+	-	-	+	-	-	-	-	20277	-
399	2021	11124	0.015	2	R	-	-	-	+	-	-	-	-	-	+	-	+	+	+	18787	G12302
400	2021	11125	0.004	0.12	S	-	-	+	-	-	-	-	-	-	+	-	+	+	+	20272	-
401	2021	11126	-	0.06	S	-	-	-	-	-	-	-	-	-	-	+	-	-	-	20292	G5042
402	2021	11127	0.004	0.12	S	-	-	-	-	-	-	-	-	-	-	-	-	-	-	20271	-
403	2021	11128	0.002	0.02	S	-	-	-	-	-	-	+	-	-	+	-	-	-	-	20268	-
404	2021	11129	0.008	0.5	S	-	-	-	+	-	-	-	-	-	+	-	+	+	+	20291	G12302
405	2021	11130	0.002	0.06	S	-	-	-	-	-	-	+	-	-	+	+	-	-	-	20290	-
406	2021	11131	0.015	2	R	-	-	-	+	-	-	-	-	-	+	-	+	+	+	18787	G12302
407	2021	11132	0.002	0.03	S	-	-	-	-	-	-	-	-	-	-	+	-	-	-	20270	-
408	2021	11134	0.002	0.12	S	-	-	-	-	-	-	+	-	+	+	-	-	-	-	1993	G1993
409	2021	11135	0.004	0.12	S	-	-	-	+	-	-	-	-	-	+	-	+	+	+	20293	G12302
410	2021	11136	0.002	0.12	S	-	-	-	-	-	-	-	-	-	-	+	-	-	-	20270	-
411	2021	11137	0.002	0.12	S	-	-	-	-	-	-	+	-	-	+	-	-	-	-	20269	-
412	2021	11139	-	0.12	S	-	-	-	-	-	-	-	-	-	+	-	-	-	-	20267	G10800
413	2021	11140	-	0.12	S	-	-	-	-	-	-	+	-	-	+	-	-	-	-	20277	-
414	2021	11141	0.008	0.12	S	-	-	-	-	-	-	-	-	-	+	-	-	-	-	20276	-
415	2021	11142	0.008	2	R	-	-	+	-	-	-	-	-	-	-	+	-	-	-	4550	-
416	2021	11143	-	1	S	-	-	-	+	-	-	-	-	-	+	-	+	+	+	20275	G12302
417	2021	11144	0.008	0.06	S	-	-	-	-	-	-	-	-	-	+	-	+	+	+	16953	G12302
418	2021	11145	0.002	0.12	S	-	-	-	-	-	-	+	-	+	+	-	-	-	-	20273	G1993
419	2021	11146	0.015	2	R	-	-	-	-	-	-	-	-	-	-	+	-	-	-	5714	G1993
420	2021	11147	0.008	0.5	S	-	-	-	-	+	-	-	+	-	+	-	-	-	-	19050	G10386

№	Year	Sample code	Ceftri-axone	Azithro-mycin		23s *rRNA*		*mtrR profile*									*mtrD profile*			NG-MAST	
			MIC_{cro} mg/L	MIC_{azm} mg/L	S/R azith	2058/ 2059	2611	−35 mosaic	−35 mosaic/ delA	−35 delA	−10 insT	Ala39 Thr	Gly45 Asp	Gly45 Ser	Ala86 Thr	His105 Tyr	mosa-ic	Ser821 Ala	Lys823 Glu	ST	Geno-group
421	2021	11148	0.004	0.25	S	-	-	-	-	-	-	+	-	-	+	-	+	+	+	5624	G5624
422	2021	11149	0.004	0.06	S	-	-	-	-	+	-	-	-	-	+	+	-	-	-	4706	G1407
423	2021	11150	0.015	1	S	-	-	-	+	-	-	-	-	-	+	-	+	+	+	20280	G758
424	2021	11151	0.008	0.5	S	-	-	-	+	-	-	-	-	-	+	-	+	+	+	20279	G12302
425	2021	11152	0.002	0.03	S	-	-	-	-	+	-	-	-	-	+	+	-	-	-	4706	G1407
426	2021	11153	0.008	0.12	S	-	-	-	-	+	-	-	-	-	+	+	-	-	-	20278	G1407
427	2021	11154	0.06	2	R	-	-	-	-	-	-	+	-	-	+	-	-	-	-	5624	G5624
428	2021	11155	0.015	0.12	S	-	-	-	-	+	-	-	+	-	+	-	-	-	-	19050	G10386
